# Vitamin B12 status and folic acid/vitamin B12 related to the risk of gestational diabetes mellitus in pregnancy: a systematic review and meta-analysis of observational studies

**DOI:** 10.1186/s12884-022-04911-9

**Published:** 2022-07-23

**Authors:** Jin He, Dongmei Jiang, Xianwei Cui, Chenbo Ji

**Affiliations:** 1 School of Nursing, Nanjing Medical University, Nanjing, China; 2 Nanjing Maternity and Child Health Care Institute, Nanjing Maternity and Child Health Care Hospital, Women’s Hospital of Nanjing Medical University, Nanjing, China

**Keywords:** GDM, Vitamin B12, Folate, Association, Meta-analysis

## Abstract

**Background:**

This review was conducted to investigate the association between serum vitamin B12 levels as well as folic acid/vitamin B12 during pregnancy and the risk of gestational diabetes mellitus (GDM).

**Methods:**

A comprehensive search of electronic databases (Embase, PubMed, and Web of Science) was performed. The odds ratios (ORs) with 95% confidence intervals (CIs) of GDM risk were summarized using a random effects model. We also performed subgroup analyses to explore the source of heterogeneity.

**Results:**

A total of 10 studies, including 10,595 pregnant women were assessed. Women with vitamin B12 deficiency were at higher risk for developing GDM when compared with those who were vitamin B12 sufficient (OR, 1.46; 95% CI 1.21–1.79; I^2^: 59.0%). Subgroup analysis indicated that this association might differ based on sample size and geographical distribution. Elevated vitamin B12 levels may decrease the risk of GDM by 23%. The role of excess folic acid and low vitamin B12 levels in the occurrence of GDM is also controversial.

**Conclusion:**

In summary, vitamin B12 deficiency is associated with increased risk of GDM, it is necessary to pay more attention to the balance of vitamin B12 and folic acid. However, more in-depth studies across multiple populations are needed to verify these results.

**Supplementary Information:**

The online version contains supplementary material available at 10.1186/s12884-022-04911-9.

## Introduction

Gestational diabetes mellitus (GDM) is one of the most prevalent complications of pregnancy. In a joint statement released in 2018, the International Federation of Obstetrics and Gynecology (FIGO) and the International Diabetes Federation (IDF) stated that high blood glucose levels during pregnancy have an impact on maternal, newborn, and child health and may contribute to the global burden of type 2 diabetes mellitus and cardiovascular metabolic disorders in the short and long term. This poses a critical public health challenge as well as a substantial financial burden [1]. As a result, effective prevention is extremely necessary.

Investigating modifiable risk factors in the early pregnancy stage would more significantly contribute to the early prevention of GDM. In current prevention and treatment programs, diet and lifestyle such as a low-glycemic index (GI) diet, Mediterranean diet and physical activity have shown the effects of reduction on GDM [2–4]. Differences in metabolic response are complex and wide, and approaches that personalize nutrition recommendations based on genetic parameters, individuals genetic, phenotypic, medical, and nutritional are promising. With the further development of nutrition-related research, several systemic reviews have shown that personalized nutrition, such as probiotics supplementation, inositol, and vitamin D can effectively reduce the risk of GDM or improve its condition [5–9].

Recently, the role of vitamin B12 and folic acid on the development of GDM has emerged as a field of interest. B vitamins are essential coenzymes in the metabolism of glucose, protein, and lipids, and are one of the vital trace nutrients during early pregnancy [10]. Vitamin B12 is a water-soluble B vitamin involved in the single-carbon unit metabolic pathway, which is negatively connected to homocysteine (Hcy) metabolism [11]. Impaired serum homocysteine metabolism, which results in microcirculation disruption and oxidative stress, might exacerbate vascular endothelial damage caused by vitamin B12 deficiency [12, 13]. It has also been shown that there is an association between hyperhomocysteinemia and insulin resistance. Low levels of vitamin B12 in pregnant rodents have been shown to change circulating microRNAs from fat sources, which may promote adipogenesis and insulin resistance [11], as well as indirectly influence blood glucose levels by taking part in insulin production, secretion, and metabolism [14, 15]. Many investigations have shown a correlation between maternal vitamin B12 levels and the development of GDM; nevertheless, studies have yielded contradictory results about the relationship between maternal vitamin B12 status and the risk of GDM. Several small cross-sectional and cohort studies published between 2009 and 2021, including the most recent 1 with 4,746 samples, found that vitamin B12 deficiency (serum vitamin B12 concentration 150–220 pmol/L) increased the risk of GDM [16–24]. In contrast, 2020 cohort research in China with 1,058 individuals discovered that high vitamin B12 levels increased the risk of GDM [25]. Considering the inconsistent results mentioned above, determining the relationship between maternal vitamin B12 status and GDM risk is urgent.

Folic acid, together with vitamins B12 as cofactors, is required to maintain Hcy levels within a normal range. Serum folic acid and vitamin B12 (folic acid/vitamin B12) imbalances are more strongly associated with GDM [26]. Folic acid is involved in DNA methylation and biosynthesis of nucleic acids and proteins required for cell replication and fetal growth and is an essential micronutrient during pregnancy [27]. Recently, only 3 systematic reviews investigated the association between vitamin B12 paired with folic acid and GDM [28–30]. Nonetheless, the findings of existing meta-analyses and systematic reviews are contradictory, and the significance of the folic acid/vitamin B12 in the risk of GDM remains unknown. A negative link was detected in 1 systematic review; however, it was based on just two publications with tiny samples [28]; the other systematic review was irrelevant as it included three completely different studies [29] and the most recent has shown that higher plasma/serum folic acid may increase GDM risk but with a low grade of quality.

Together, these factors make it necessary to determine the role of maternal vitamin B12 status as well as combined with the status of folic acid in GDM development. In this meta-analysis and systematic review, we aimed to clarify the relationship between the risk of GDM and serum vitamin B12, with the folic acid/vitamin B12, and to provide the evidence for further exploration of the role of trace elements in GDM.

## Materials and methods

This study was conducted in accordance with Cochrane’s Preferred Reporting Items for Systematic Reviews and Meta-Analyses 2020 (PRISMA).

### Search strategy

A systematic literature search was performed in the Embase, PubMed, and Web of Science databases on June 5, 2022. The following combination of keywords was used to identify studies from the electronic database: (“Vitamin B 12”[MeSH Terms] OR “Cobamides”[MeSH Terms] OR “Vitamin B 12 Deficiency”[MeSH Terms]) AND (“diabetes, gestational”[MeSH Terms] OR “Pregnancy in Diabetics”[MeSH Terms] OR “gestational diabetes mellitus”[All Fields]) in PubMed; Topic = (“gestational diabetes” or gdm or “gestational diabetes mellitus” or “diabetes in pregnancy”) AND Topic (“vitamin b12” or cobalamin or cyanocobalamin or methylcobalamin or “b12 vitamin”) in Web of Science; and TX (“gestational diabetes” or gdm or “gestational diabetes mellitus” or “diabetes in pregnancy”) AND TX (“vitamin b12” or cobalamin or cyanocobalamin or methylcobalamin or “b12 vitamin”) in Embase. The reference lists of retrieved articles and relevant reviews were also manually searched. The search was restricted to articles published in English.

### Inclusion and exclusion criteria

**Fig. 1:**
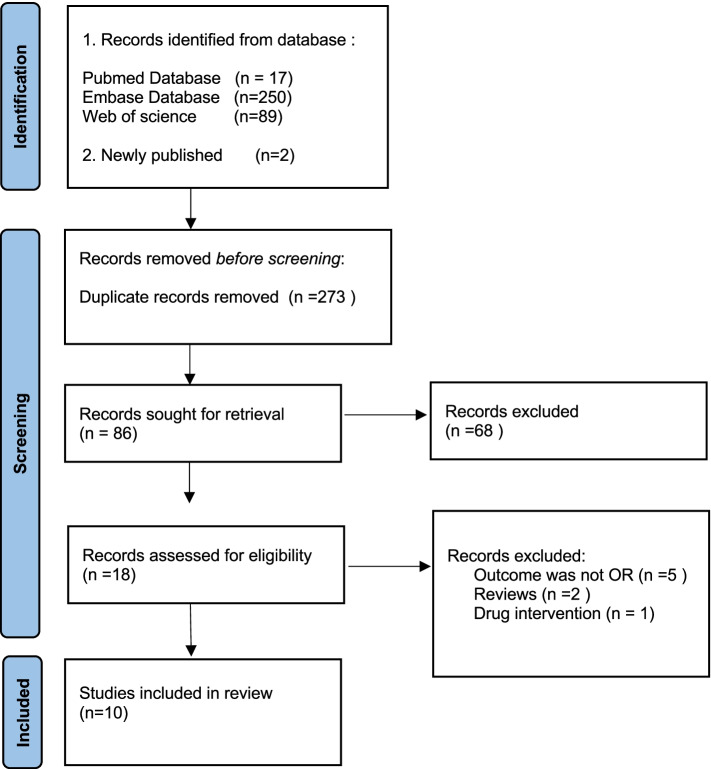
PRISMA flow chart of the study identification process

All of the published articles included in the analysis were matched to the following criteria:⑴Patients with clinically diagnosed GDM during pregnancy; ⑵ observational studies evaluated the association between vitamin B12 status or folic acid/vitamin B12 and prevalence or incidence of GDM, serum vitamin B12 concentration ≤ 220 pmol/L is considered vitamin B12 insufficiency; ⑶ the study reported effect estimate [risk ratio (RR), hazard ratio (HR), or odds ratio (OR)] and corresponding 95% confidence interval (CI) for comparisons of sufficient vitamin B12 and insufficient vitamin B12 levels. Studies were excluded if ⑴ the article did not provide full text or contained insufficient information for assessment; ⑵ extra supplements of vitamin B12 before GDM diagnosis; ⑶ conference paper;(4) papers that reported the same data from the same study populations. The selection process and reasons for exclusion are presented in Fig. 1.

### Data collection and quality assessment

Two reviewers (Jin He and Dongmei Jiang) independently screened titles and abstracts for full-text review, duplicate entries were removed, and any disagreements between the two authors were settled by group discussion until a consensus was reached. The collection of the data included author, year, country, study design, sample size, assessment of vitamin B12 and folic acid/vitamin B12 levels, GDM diagnostic criteria and time, and the effect estimate with 95% CI. Study quality was assessed according to the Newcastle–Ottawa Scale (NOS) [31, 32]. NOS guidelines include three quality parameters: four items for selection, two items for comparability, and three items for outcomes. Studies scoring 7–10 were identified as high-quality, those with scores of 3–6 were considered moderate quality, and the others were of low quality. In the present study, studies that achieved six or more stars were considered high quality.

### Statistical analysis

Statistical analysis was performed on Stata software (Version 16.0), and the OR and 95% CI were used for the dichotomous variable [32]. From the perspective of the calculation formula, when the incidence of study outcomes is rare, RR could be approximately instead of OR [33]. The heterogeneity among all studies was assessed by the Q test and I^2^ statistics. If the I^2^ ≥ 50%, heterogeneity is considered high and a random-effects model is used. Sensitivity analysis was also performed by excluding each study one by one to evaluate the credibility of pooled results. To identify sources of heterogeneity, we also used stratified analysis, including geographic location, trimester, GDM diagnostic criteria, gestational weeks for detecting serum vitamin B12 and folic acid levels, and the number of samples.

### Evidence quality assessment

The certainty of evidence from cohort studies was assessed using NutriGrade shown in the Supplementary Table 2 [34]. The NutriGrade scoring system includes 8 items for meta-analyses of cohort studies as follows:1) risk of bias, study quality, and study limitations, 2) precision, 3) heterogeneity, 4) directness, 5) publication bias, 6) funding bias, 7) effect size, and 8) dose response. 4 categories were recommended to judge the meta-evidence: ≥ 8 points (high meta-evidence); 6–7.99 points (moderate meta-evidence); 4–5.99 points (low meta evidence); and 0–3.99 points (very low meta-evidence).

## Results

Figure 1 displays an integrated flow diagram of the identification and recognition process of the involved studies. A total of 357 citations were originally retrieved by searching PubMed, Embase, and Web of Science; none of the studies were manually identified by reviewing the reference list of the relevant literature. 84 citations remained after removing duplicate articles. After screening the titles and abstracts, 68 articles were excluded. Finally, during the completion of the study, 2 new articles were retrieved, and 9 of the remaining 17 articles that examined the relationship between vitamin B12 status and the risk of GDM were included in this quantitative synthesis after careful reading and review. Of the 10 articles, 7 explored the risk relationship between folic acid/vitamin B12 and GDM.

### Study characteristics

The baseline characteristics of the eligible studies are presented in Table 1. Among the 10 studies, there were 4 prospective cohort studies with sample sizes ranging from 180 to 4,746 [17, 24, 25, 35], 2 were cross-sectional studies including a total of 1,319 individuals [18, 26], and 4 were case–control studies [22, 23, 27, 36]. Of the 10 studies, 5 were performed in China [18, 23, 25–27], 3 in India [17, 18, 35], 1 in Australia [36], and two in Britain [22, 24]. The cutoff values of vitamin B12 were not uniform and included studies that primarily used 150 pmol/L–220 pmol/L. The NOS was used to assess the quality of studies, and scores ≥ 6 were considered high quality. 8 of the studies had a score of 7 [17, 18, 22, 24–27, 36], and the remaining 2 studies had a score of 4 [23, 35]. Regarding the GDM criteria, 5 studies used the International Association of Diabetes and Pregnancy Study Groups (IADPSG) [23–27], 3studies used the WHO1999 [17, 18, 22], 1used the WHO2016 criteria [36], and 1 was based on the criteria of Carpenter and Coustan [35]. The quality of the included literature was medium, and the results were shown in Supplementary Table 1.

**Table 1:** Characteristics of studies investigating the association between vitamin B12 and gestational diabetes mellitus (*N* = 10, from 2007–2022)

Study	Country	Study design	Sample size	Trimester of Vitamin B12	GDM diagnosis	Vitamin B12 and GDM			Folic acid/Vitamin B12 and GDM			Score
						**OR**	**95%CI**	***P*-value**	**OR**	**95%CI**	***P*-value**	
Wang, 2020 [23]	China	case–control	NR^1^	Third trimester	IADPSGC^2^	1.31	1.03–1.68	0.03	-	-	-	4
Sukumar, 2016 [22]	British	Retrospective case–control	344	Second trimester	WHO 1999^3^	2.59	1.35–4.98	0.004	-	-	-	7
Krishnaveni, 2009 [17]	India	Prospective cohort-study	785	Third trimester	WHO 1999^3^	2.00	1.1–3.6	0.02	-	-	-	7
Chen, 2021 [25]	China	Prospective cohort-study	1058	First trimester	IADPSGC^2^	1.54	1.41–1.68	0.002	0.98	0.97–0.99	0.009	7
Li, 2019 [26]	China	Cross-sectional	406	Third trimester	IADPSGC^2^	0.30	0.15–0.60	< 0.05	3.08	1.63–5.83	< 0.005	7
Lai, 2018 [18]	China, India, Malaysia	Cross-sectional	913	Second trimester	WHO 1999^3^	0.81	0.68–0.97	0.02	1.97	1.05–3.68	0.034	7
Tan, 2020 [36]	Australia	Nested-cohort	325	Second trimester	WHO 2016^4^	0.99	0.83–1.18	0.913	1.37	0.71–2.62	0.347	7
Sukumar, 2021 [24]	British	Prospective cohort-study	4746	First trimester	IADPSGC^2^	0.86	0.79–0.93	< 0.05	1.74	1.23–2.44	0.003	7
						1.20	1.00–1.44	0.05				
Krishnaveni, 2007 [35]	India	Prospective cohort-study	654	Third trimester	Carpenter-Coustan criteria^5^	1.90	0.98–3.68	0.057	4.8	1.45–15.84	0.01	4
Li, 2022 [27]	China	case–control	1364	Second trimester	IADPSGC^2^	0.80	0.65–0.98	0.033		-	-	7

^1^NR, Not Report

^2^IADPSGC, International Association of Diabetes and Pregnancy Study Groups (75 g OGTT, fasing > 5.1 mmol/L, 1-h > 10.0 mmol/L, 2-h > 8.5 mmol/L)

^3^WHO 1999, 1999 World Health Organization standard criteria (75 g OGTT, fasting > 7.0 mmol/L; 2-h > 7.8 mmol/L)

^4^WHO 2016, the new (2016) World Health Organization classification (fasting ≥ 5.1 mmol/L 2-h level of ≥ 8.5 mmol/L)

^5^Carpenter-Coustan (50 g OGTT, fasting > 5 mmol/L, 1-h > 10.0 mmol/L, 2 -h > 8.6 mmol/L, 3 -h > 7.7 mmol/L)

### Association between vitamin B12 and GDM

**Fig. 2:**
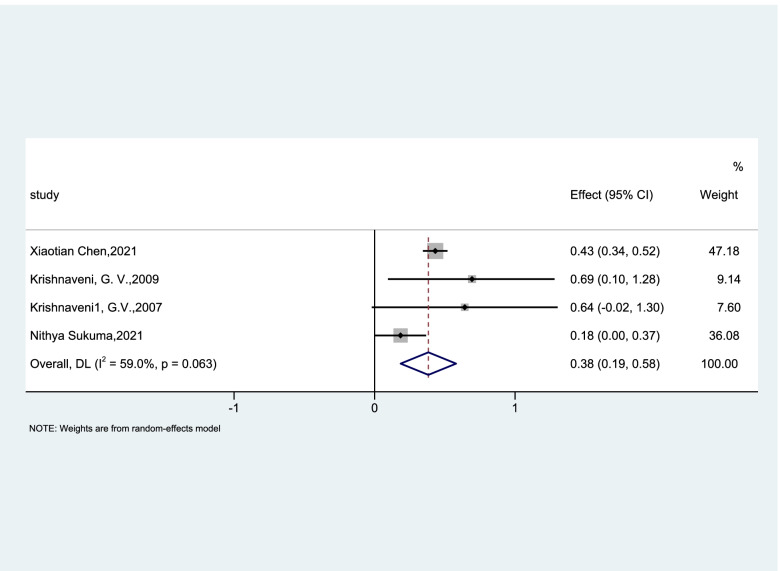
Forest plots (random effects model) of meta-analysis of cohort studies on the association between the concentration of vitamin B12 and the risk of GDM. The data in the graph are logarithmically converted

**Fig. 3:**
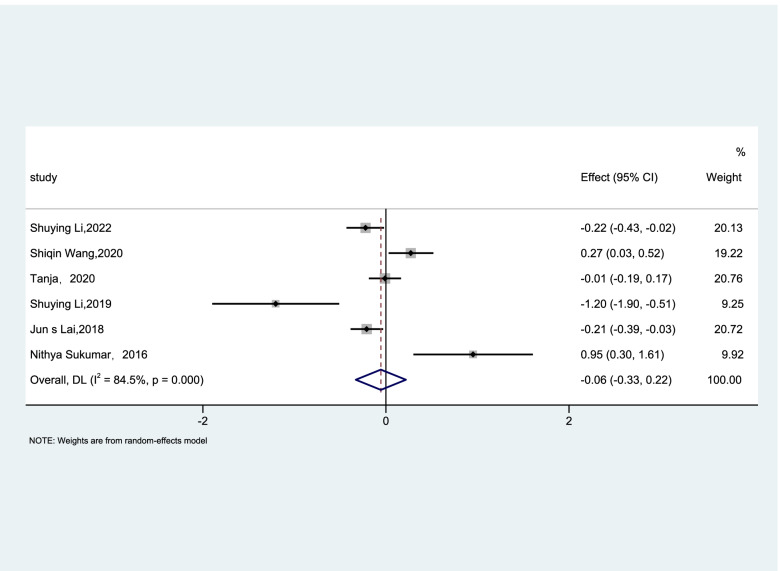
Forest plots (random effects model) of meta-analysis of cross-sectional studies on the association between the concentration of vitamin B12 and the risk of GDM. The data in the graph are logarithmically converted

To explore the link between vitamin B12 and GDM, 4 prospective cohort studies [17, 24, 25, 35] were included in the meta-analysis to determine the relationship between vitamin B12 status and the risk of GDM. 4 studies all reported that vitamin B12 deficiency may increase the risk of GDM, and the OR of the random effects model was 1.46 (95%CI, 1.21–1.79, I^2^ = 59%, *P* = 0.063; Fig. 2). 2 cross-sectional studies showed that high levels of vitamin B12 may reduce the risk of GDM [18, 26] and 4 case–control studies had different results. We included these 6 studies in a random effects model and the OR was 0.97(95%CI, 0.76–1.25, I^2^ = 81.6%, *P* < 0.001; Fig. 3) indicating that vitamin B12 concentrations have no significant association with the incidence of GDM. Sensitivity analysis was conducted after each study was eliminated and revealed that the heterogeneity was not improved.

### Association between folic acid/vitamin B12 ratio and GDM

**Fig. 4:**
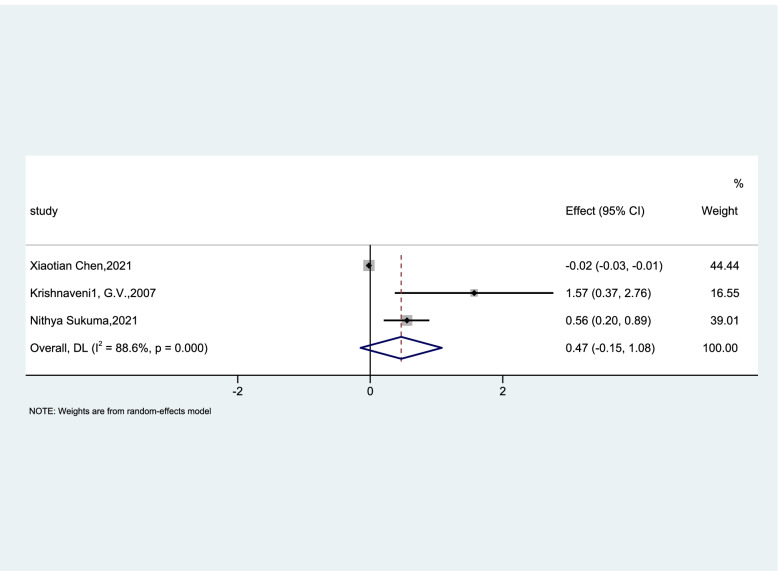
Forest plots (random effects model) of meta-analysis of cohort studies on the association between the between folic acid/vitamin B12 ratio and GDM. The data in the graph are logarithmically converted

**Fig. 5:**
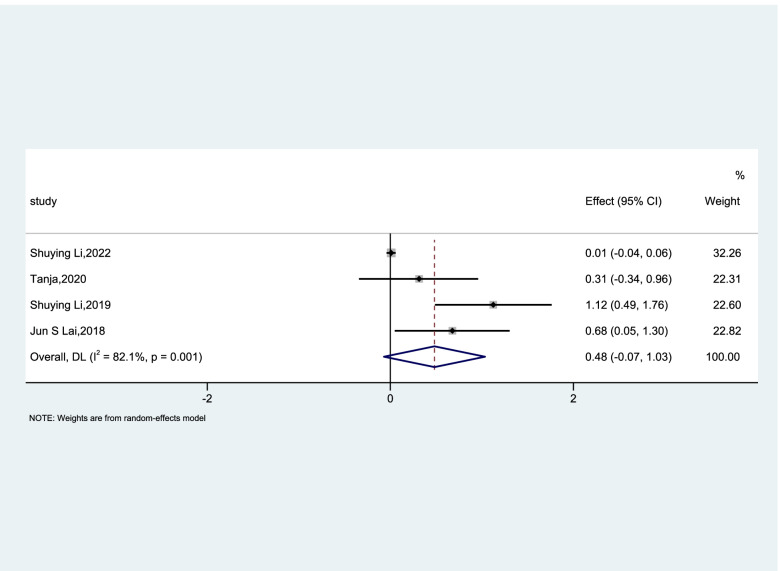
Forest plots (random effects model) of meta-analysis of cross-sectional studies on the association between the between folic acid/vitamin B12 ratio and GDM. The data in the graph are logarithmically converted

Utilizing dietary micronutrient ratios in nutritional research may be more informative than focusing on single nutrients. To observe whether the ratio of two trace elements is related to GDM, we collected data on the folic acid/vitamin B12, and the risk of GDM studied in the literature. We summarized the OR value from 7 articles. Only Chen et al. [25] indicated that greater serum floated/vitamin B12 ratio was associated with decreased GDM risk (OR, 0.98; 95% CI 0.97–0.99; *P* = 0.009). However, Li et al. [26] (OR, 2.61; 95% CI 1.44–4.75; *P* = 0.002), Lai et al. [18] (OR, 1.97; 95% CI 1.05–3.68; *P* = 0.034), Krishnaveni et al. [35] (OR, 4.8; 95% CI 1.45–15.84; *P* = 0.01), and Sukumar et al. [24] (OR, 1.74; 95% CI 1.23–2.43; *P* = 0.003) reported inverse results. Finally, the result of cohort studies showed that insufficient vitamin B12 with excess folic acid could increase the risk of GDM by 60% (OR, 1.60; 95% CI 0.86–2.94; *p* < 0.001; I^2^ = 88.6%; Fig. 4). We also analyzed cross-sectional studies in a random-effects model and the OR was 1.63 (95% CI 0.93–2.80; *p* = 0.001; I^2^ = 82.1%; Fig. 5) showing that there was no evidence that the ratio of vitamin B12 to folic acid was associated with the risk of GDM.

### Subgroup analysis

To explore the sources of high heterogeneity in the included studies, several subgroup analyses were carried out which contained geographic location, diagnosis of GDM, levels of vitamin B12, gestational weeks for detecting serum vitamin B12 and folic acid levels, and the number of samples. The results of all stratified analyses by study characteristics are shown in Table 2. When the data were stratified by geographic location, there was indicated that exposure to vitamin B12 deficiency is more likely to develop GDM than those with normal vitamin B levels by 95% in South Asian (95%CI 1.26–3.03, I^2^ = 0%, *P* = 0.91). When stratified by GDM diagnosis and the trimester of vitamin B12 measurement, in the first trimester, vitamin B12 deficiency was associated with an increased risk of GDM diagnosed by IADPSGC (OR, 1.34; 95%CI 1.08–1.75). Unsurprisingly, in the subgroup analysis of the cross-sectional study and the number of samples, the results showed that high levels of vitamin B12 in serum are protective factors for GDM (OR, 0.77; 95% CI 0.63–0.94; OR, 0.82; 95%CI 0.72–0.93).

**Table 2:** Subgroup analysis of the risk of gestational diabetes mellitus and vitamin B12

	**No. studies**	**Summary OR** **(95% CI)**	**I**^**2**^**(%)**	P*	P**	P***
Cohort studies	4	1.46(1.21–1.79)	59.0%	< 0.001	0.063	-
Subgroup						
Country						
European	1	1.20 (1.00–1.45)	-	-	0.046	0.03
Asia	1	1.54 (1.41–1.67)	-	-	< 0.00	
South Asia	2	1.95(1.26–3.03)	0%	0.91	0.003	
Trimester						
First	2	1.38(1.08–1.75)	82.6%	0.016	0.009	0.0039
Second	1	1.99 (0.98–3.63)	-	-	0.057	
Third	1	1.90 (0.98–3.67)	-	-	0.022	
GDM diagnosis						
IADPSGC	2	1.38(1.08–1.75)	82.6%	0.016	0.009	0.0031
WHO1999	1	1.90 (0.98–3.67)	-	-	0.022	
Carpenter-Coustan criteria	1	1.99 (0.98–3.63)	-	-	0.046	
Cross-sectional studies	6	0.97(0.76–1.25)	81.6%	< 0.001	0.691	
Subgroup						
Vitamin B12 levels						
high	3	0.77 (0.63–0.94)	48.1%	0.145	0.011	
low	3	1.33 (0.91–1.94)	79.6%	0.007	0.138	
Sample size						
> 500	2	0.82 (0.72–0.93)	0%	0.786	0.002	0.191
< 500	4	1.02 (0.72–1.67)	84.0%	< 0.001	0.666	
Country						
European	2	1.52 (0.59–3.86)	87.1%	0.005	0.382	0.249
Asia	4	0.85 (0.64–1.14)	81.8%	0.001	0.285	
GDM diagnosis						
IADPSGC	3	1.83 (0.51–1.34)	87.1%	< 0.001	0.447	0.648
WHO1999	2	1.44 (0.46–4.26)	90.9%	0.001	0.552	
WHO2016	1	0.90 (0.83–1.18)	-	-	0.911	
Trimester						
First	1	0.90(0.83–1.81)	-	-	0.911	0.107
Second	4	0.88(0.61–1.27)	81.2%	0.001	0.502	
Third	1	1.31(1.03–1.68)	-	-	0.030	

P*-value for heterogeneity of subgroup. P**-value for tests of subgroup effect size. P**-value for Q statistics between-subgroup heterogeneity

### Evidence quality assessment

The NutriGrade scoring system had a score of 6.25, which indicated a low level of meta-analysis evidence (Supplementary Table 2). Further research may provide important evidence on the confidence and likely change the effect estimate.

## Discussion

This comprehensive quantitative meta-analysis was based on 10 observational studies involving 10,595 pregnant women, and the result of cohort studies revealed that maternal B12 insufficiency was associated with an increased risk of GDM by 46% (95% CI, 1.21–1.79). But the result of cross-sectional studies and case controls displayed that there was no distinct association between vitamin B12 levels and the risk of GDM (OR, 0.97; 95%CI, 0.76–1.25). Additionally, we also found that the imbalance of serum folic acid and vitamin B12 (folic acid/vitamin B12) was not significantly associated with GDM. This contradicts previous studies showing that an imbalance of serum folic acid and vitamin B12 increases the incidence of GDM.

The previous meta-analysis suggested vitamin B12 deficiency could increase the risk of GDM which was consistent with our findings [28]. However, the previous meta-analyses had some limitations that could be improved. For example, lack of available studies, a relatively small number of studies was included in this meta-analysis. Furthermore, the latest meta-analysis indicated that the association between the risk of GDM and vitamin B12 deficiency is conflicting [29], and the conclusion was derived from descriptive analysis which does not allow to draw safe conclusions. Due to these contradictory results, we conducted this updated analysis that included all published observational studies to clarify the potential link between vitamin B12 levels and imbalance of serum folic acid and vitamin B12 to the risk of GDM.

A large number of research not only allowed us to determine the relationship between vitamin B12 and the risk of GDM but also carry out huge heterogeneity which needs stratify data and sensitivity analyses to evaluate. Based on subgroup analyses, a significant association was observed between deficiency in vitamin B12 levels and an increased risk of GDM, sufficient vitamin B12 concentration may decrease GDM risk by 23% (OR, 0.77; 95% CI, 0.63–0.94). As regards geographical differences, India showed a high rate of vitamin B12 deficiency in pregnant women, which increases the risk of GDM by 95% (OR, 1.95; 95% CI 1.26–3.03). Folic acid and vitamin B12 interweave during carbon metabolism, we also found that the existing studies focused on vitamin B12 and folic acid imbalance, therefore, the association between the folic acid/vitamin B12 ratio and GDM was analyzed and found that there was no significant association in either type of study. In light of the above findings, the relationship between nutrients and GDM is still controversial, it is necessary to further study the role of a balanced relationship between folic acid and vitamin B12 in disease.

Our main findings were consistent with the major results of previously published studies. Krishnaveni et al. [35] conducted a cohort study in India and found that women with GDM had lower vitamin B12 status. However, after adjusting for several important confounding factors such as BMI and socioeconomic status, there was no significant association between decreased vitamin B12 levels and increased risk of GDM. Wang et al. [23], Sukumar et al. [22] and Krishnaveni et al. [17] conducted case–control and cohort studies with blood samples from 1,129 pregnant women drawn in mid-late pregnancy and showed that maternal vitamin B12 insufficiency increased the risk of GDM. In other terms, higher levels of vitamin B12 reduced the risk of GDM [18, 26, 27]. In contrast, Chen et al. [25] performed a prospective cohort study in China during the first trimester and indicated that women with high levels of vitamin B12 may be at an increased risk of GDM; however, a cohort study involving more than 4,000 participants in the Britain revealed an increased risk due to vitamin B12 deficiency in early pregnancy [24].

Considering that the sample size and the diagnostic criteria affect the overall combined results, based on the subgroup analyses, we found sufficient vitamin B12 levels were related to protecting the effect on the decreased occurrence of GDM when come to numerous sample sizes. Given the regional differences in diet and physical properties of vitamins, pregnant women in countries like India, which mostly includes plant-based foods and less meat than is typical in European diets, because vitamin B12 widely occurs in animal-based food products people are vegetarian may be more likely to develop GDM. Moreover, it has been demonstrated that serum vitamin B12 content is dynamic throughout pregnancy, with the highest level in the first trimester decreasing gradually to the lowest level in the third trimester [37, 38]. As pregnancy progresses, the level of estrogen in the patient’s body significantly increases, and purine metabolism increases, leading to increased consumption of folic acid and vitamin B12, which disrupts the balance of nutrients in the body. Further, the enlarged uterus puts pressure on the lumen, which reduces gastrointestinal motility and the absorption of folic acid and vitamin B12. It is important to note that studies have shown a significant decrease in vitamin B12 levels in diabetics treated with metformin [39]. Thus, we recommend those women in South Asia may require vitamin B12 testing and moderate supplementation in the first trimester of pregnancy, especially for the patients who are in need of metformin therapy.

Although an imbalance of folic acid and vitamin B12 was not observed to be associated with GDM, epidemiological studies have shown that the combination of high folic acid and low vitamin B12 is associated with the onset of GDM and adverse outcomes in offspring [40], potentially through exacerbating B12 deficiency [41]. The mechanism by which high folic acid/low B12 status is associated with an increased risk of GDM is unclear. Methyl traps that lead to elevated homocysteine levels and impaired methylation responses and altered mitochondrial metabolism may be contributing factors [42]. Longitudinal cohort studies and vitamin B12 supplementation trials are needed to confirm the association between GDM risk and folic acid/vitamin B12 status to determine the optimal dose of folic acid and vitamin B12 to achieve a “metabolic balance” of both vitamins throughout pregnancy.

Despite the heterogeneity of our results, the quality of evidence is not very excellent, we just gave a relative result and provided a synthetic and comprehensive review. However, vitamin B12 still plays a significant role in the development of pregnancy-related disorders from a clinically significant perspective. As an essential microelement involved in DNA methylation, amino acid, nucleic acid, and lipid synthesis, a growing number of cohort studies have found that B12 insufficiency is associated with sleep disturbances [43], increased amniotic fluid [44], preeclampsia [45], and liver damage [46], which are all linked to glucose and lipid metabolism. Pregnant women at high risk of vitamin B12 deficiency include those with anemia, hyperlipemia, a history of gastrectomy, and poor dietary and sleep patterns. For this group of people, in addition to evaluating objective biochemical indicators, medical personnel should evaluate daily dietary nutrient intake and nutrient supplements to maintain the balance of vitamins in the body. Simultaneously, we found that excess folic acid and low vitamin B12 levels may play a potential role in the development of GDM. This highlights the need for a further study of the appropriate content and ratio of vitamin supplements during pregnancy.

Advantages of our study include those 10 studies were included, all OR values were combined which is more than in previous studies, and the subgroup analysis by time to detect serum vitamin content, along with an investigation of the relationship between folic acid/vitamin B12 and the risk of GDM was carried out for the first time.

However, we still need to acknowledge that this review has several limitations, which means that our results should be interpreted with caution. First, the potential confounding factors in several studies could not be completely ruled out and such differences could have influenced the outcomes of individual studies. Second, data on dietary vitamin B12 intake, the use of additional vitamin B12 supplements, and nutritional habits of the participants were not available or reported by most of the included studies which may affect the concentration of vitamin B12. Third, the different diagnostic criteria used may also affect the prevalence of GDM to some extent, this was one cause of the large heterogeneity among articles. Last, half of the studies were cross-control and case reports, the analysis used pooled data (as individual data were not available), which restricted us from performing a more detailed relevant analysis and obtaining more comprehensive results. Therefore, more high-quality cohort studies are needed to verify the relationship between vitamin B12 as well as folic acid/vitamin B12 ratio and GDM.

## Conclusions

In conclusion, vitamin B12 deficiency plays an important role in the risk of GDM, especially during the first trimester in South Asian women. Folic acid/vitamin B12 has the potential ability to be a sensitive index to evaluate the relationship between nutrients and GDM. We recommend routine screening of pregnant women for vitamin B12 deficiency and folic acid excess to determine whether supplements of vitamin B12 and folic acid should be administered to ensure the nutritional status and to improve the health of the maternal. There is a need for high-quality research involving different geographical areas and ethnic groups to add credibility to the available evidence.

## Supplementary Information

**Additional file 1:** **Supplementary Table.** 1-1 Newcastle–Ottawa Scale of cohort studies.

**Additional file 2: Supplementary Table. 2. **Assessment of the Meta-Evidence of Cohort Studies for NutriGrade.

**Additional file 3: Supplementary Table.** 3 PICOS framework.

## Data Availability

The data described in this article can be freely and openly accessed from the original published articles in the database.

## Abbreviations

GDM: Gestational diabetes mellitus
OR: Odd ration
CI: Confidence interval
FIGO: International Federation of Obstetrics and Gynecology
IDF: International Diabetes Federation
Hcy: Homocysteine
PRISMA: Preferred Reporting Items for Systematic Reviews and Meta-Analyses
NOS: Newcastle-Ottawa Scale
IADPSG: International Association of Diabetes and Pregnancy Study Group
