# Supplementary material for: Vitamin B12 status and folic acid/vitamin B12 related to the risk of gestational diabetes mellitus in pregnancy: a systematic review and meta-analysis of observational studies
Source: BMC Pregnancy Childbirth. 2022 Jul 23;22:587. doi: 10.1186/s12884-022-04911-9 (PMC9308279; doi:10.1186/s12884-022-04911-9)
Supplement: Supplementary file 1 — Additional file 1: Supplementary Table. 1-1 Newcastle–Ottawa Scale of cohort studies. [file 12884_2022_4911_MOESM1_ESM.docx]

**Supplementary Table. 1-1 Newcastle–Ottawa Scale of cohort studies**

| Author | Representativeness of exposed cohort | Selection of the non-exposed  cohort | Ascertainment  of exposure | Outcome of interest was not present | Comparability of exposure and  non-exposure | Ascertainment  of outcome | Follow up enough for outcome | Adequacy of follow up |
| --- | --- | --- | --- | --- | --- | --- | --- | --- |
| G.V.Krishnaveni1, 2007 | 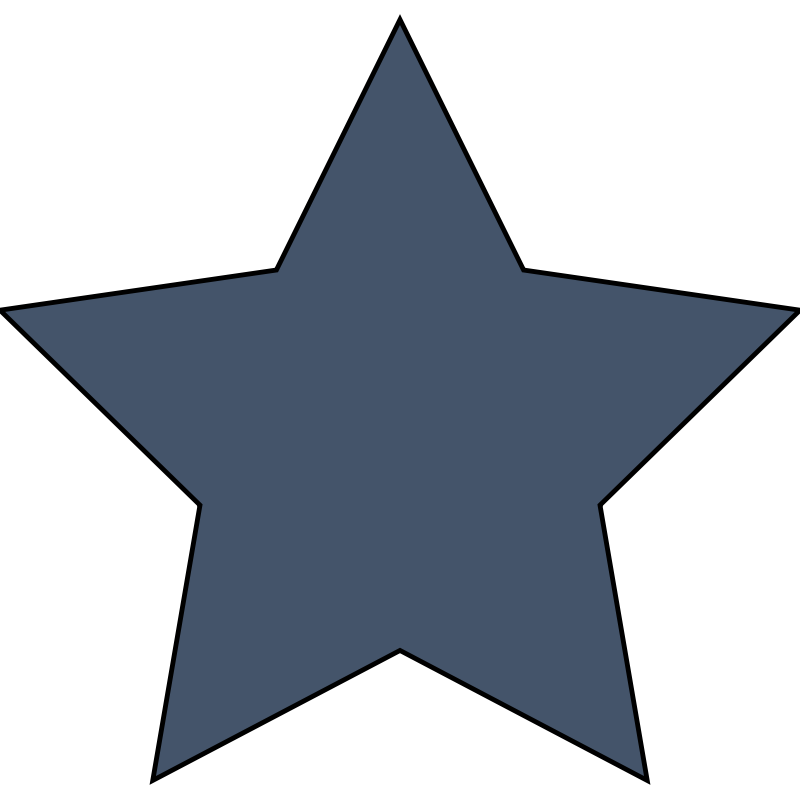 |  |  |  | 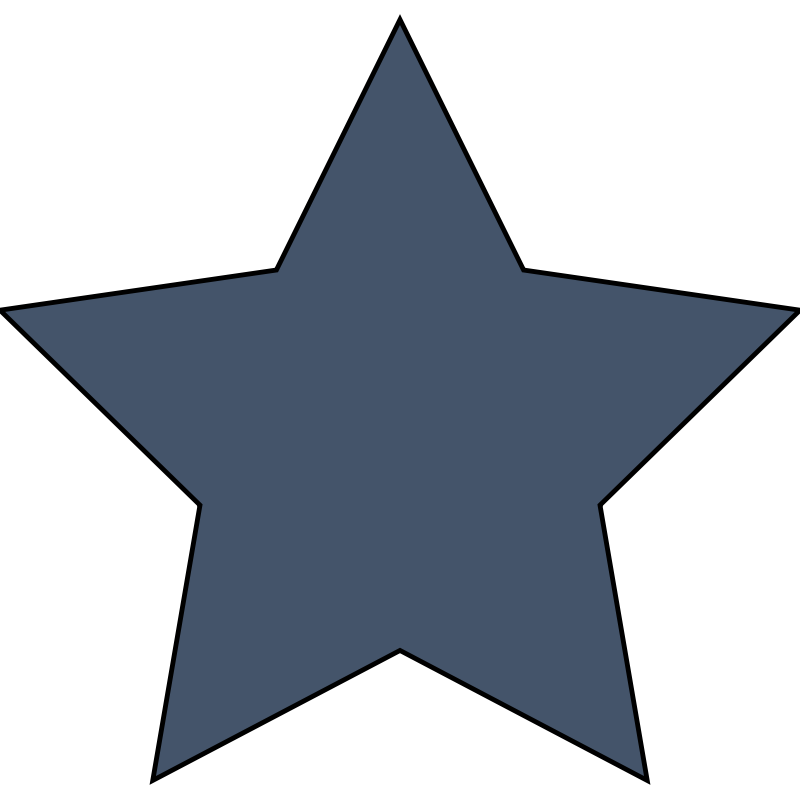 | 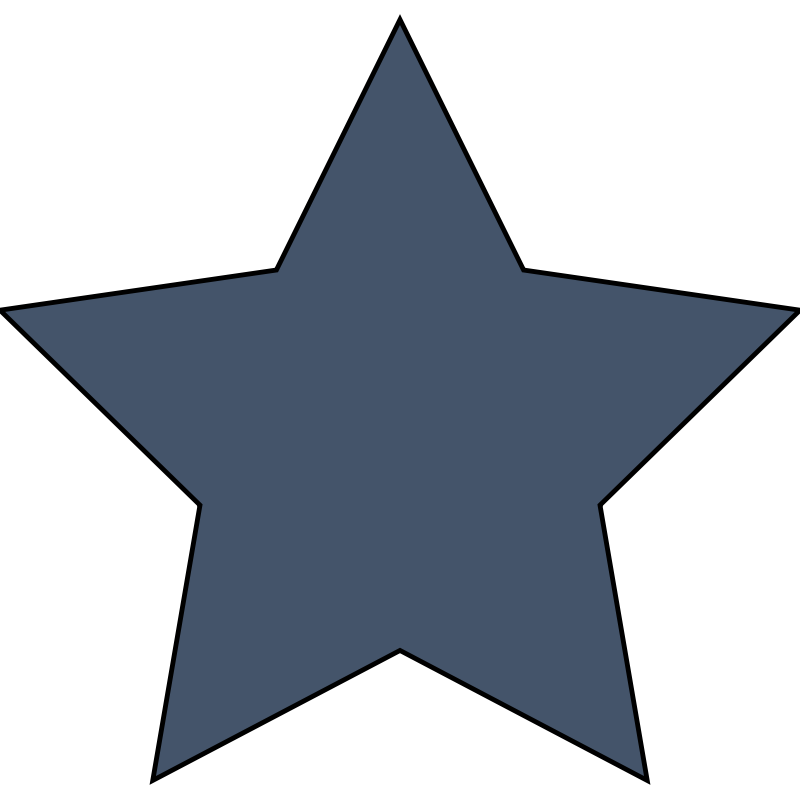 | 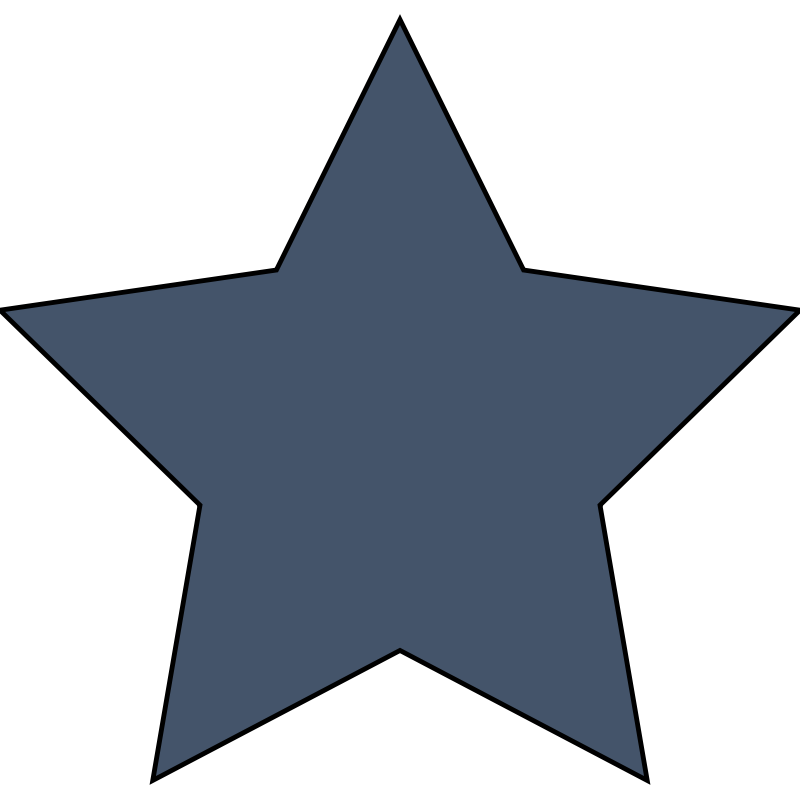 |  |
| G.V.Krishnaveni1, 2009 | 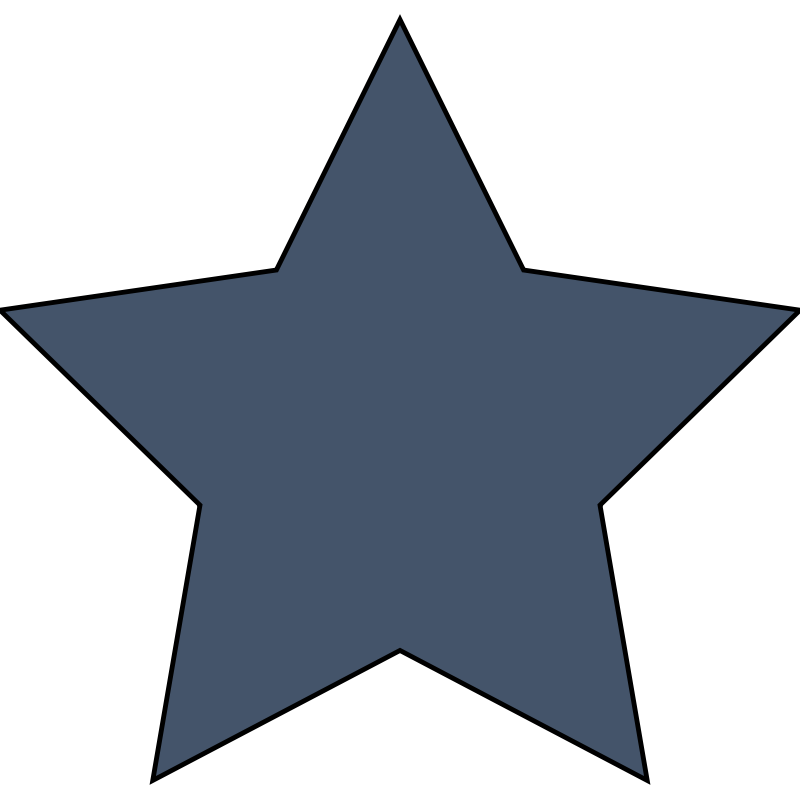 | 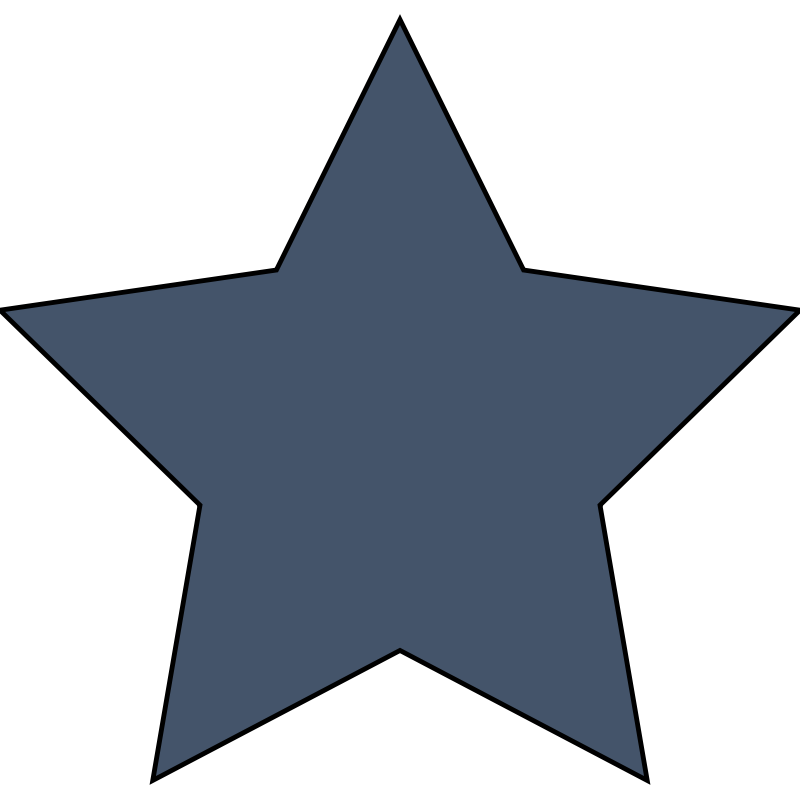 | 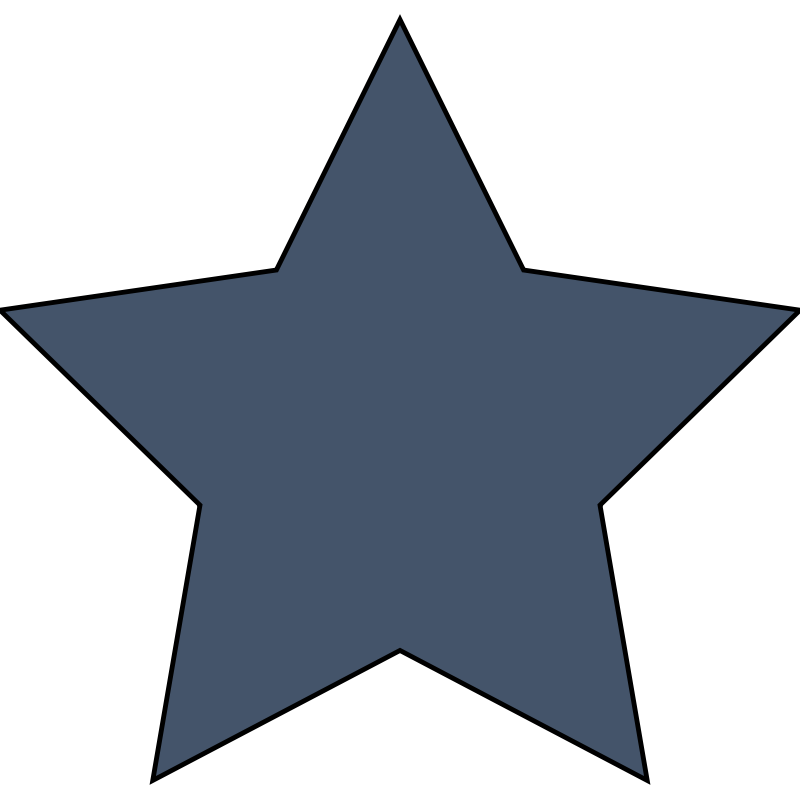 |  | 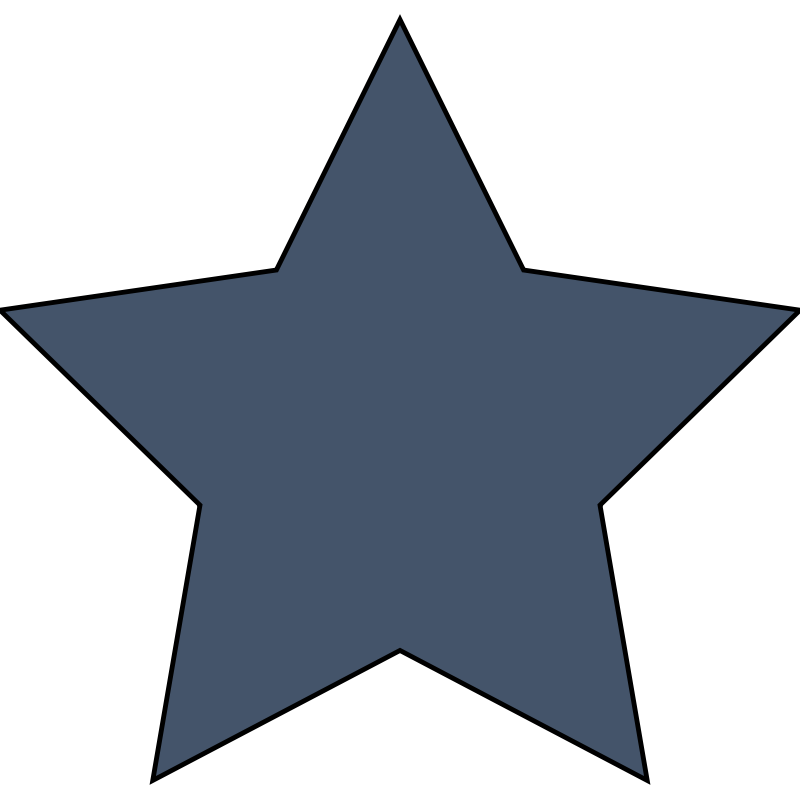 | 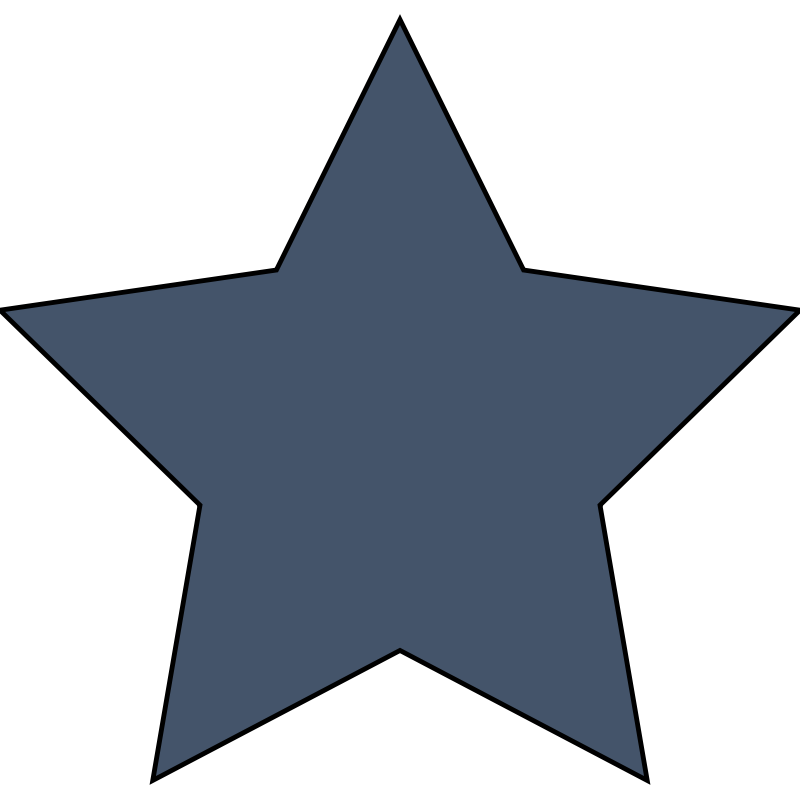 | 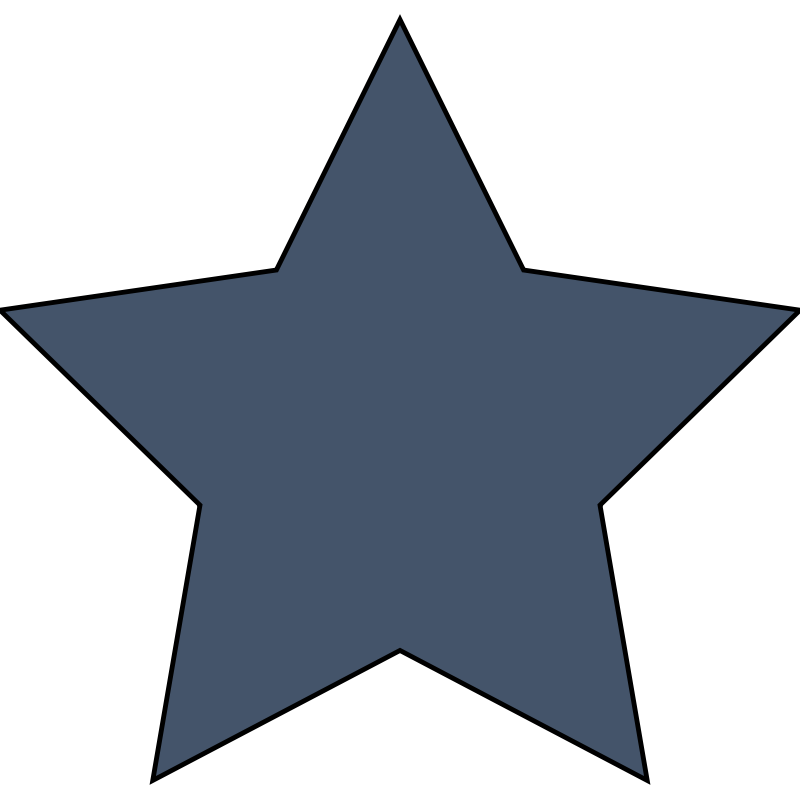 | 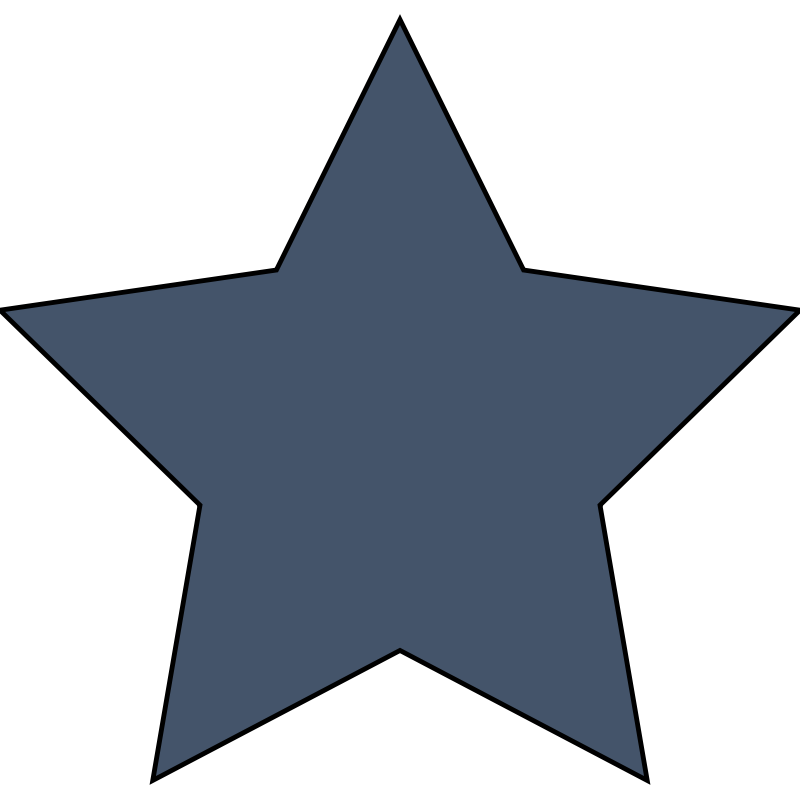 |
| Xiaotian Chen, 2019 | 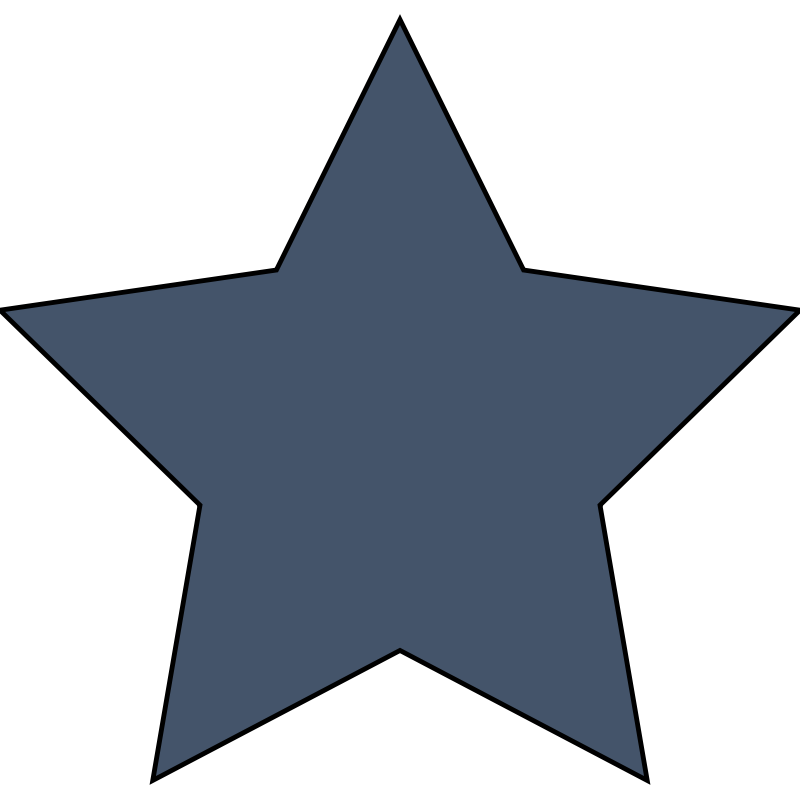 | 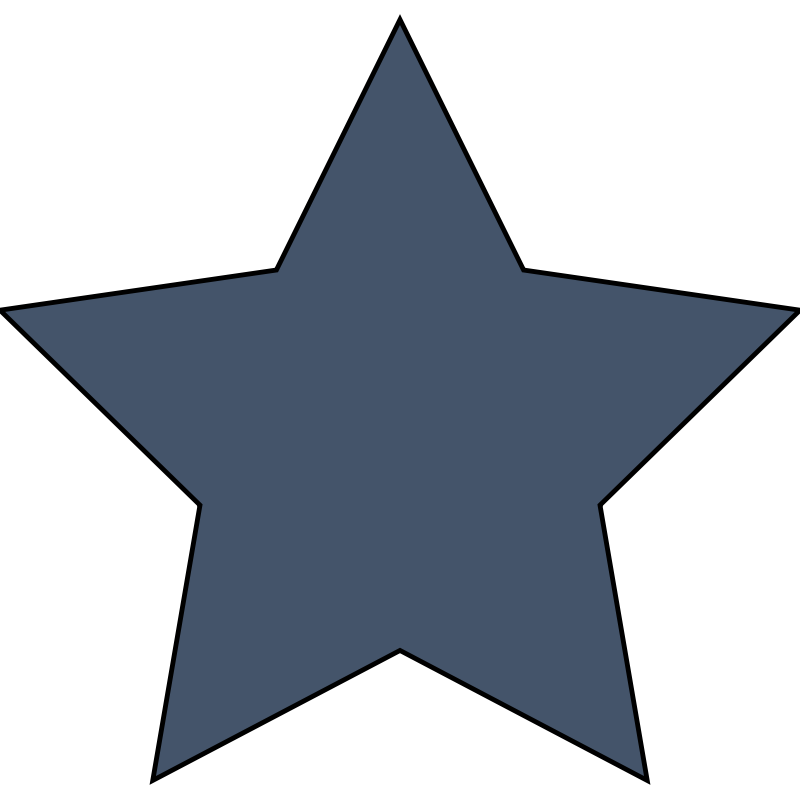 | 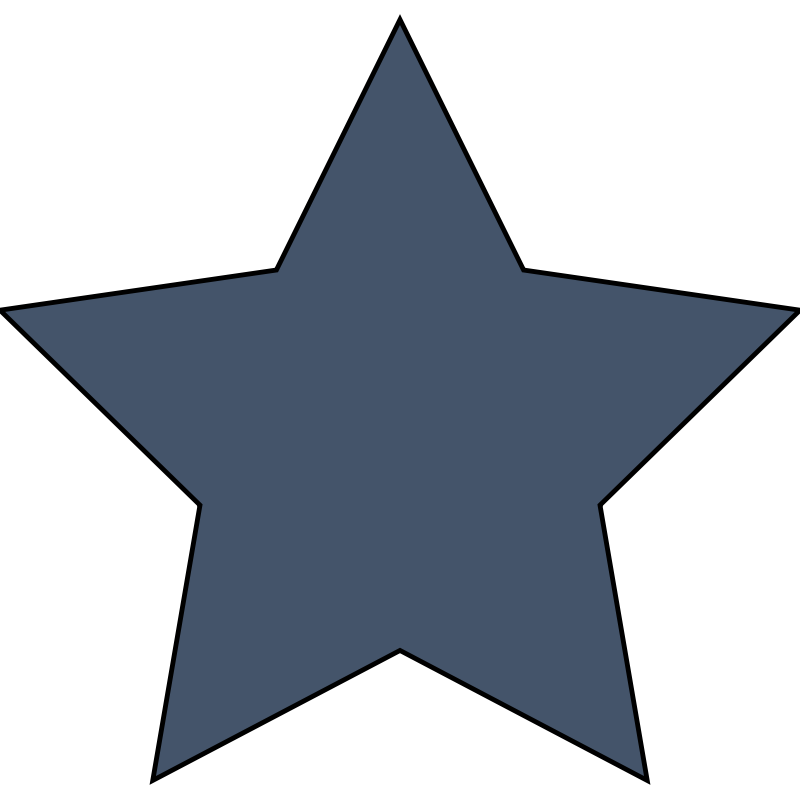 |  | 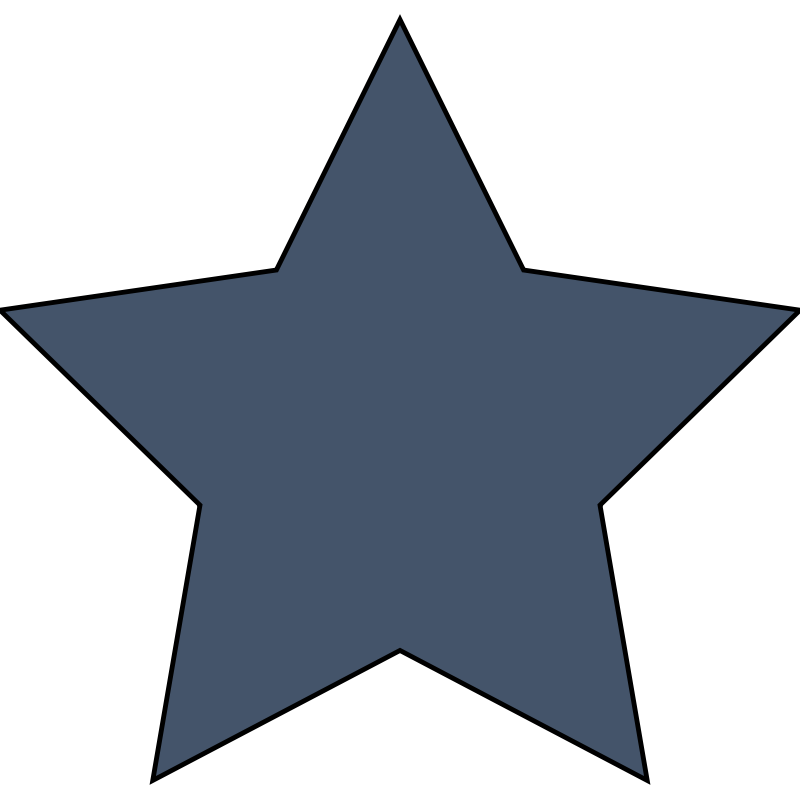 | 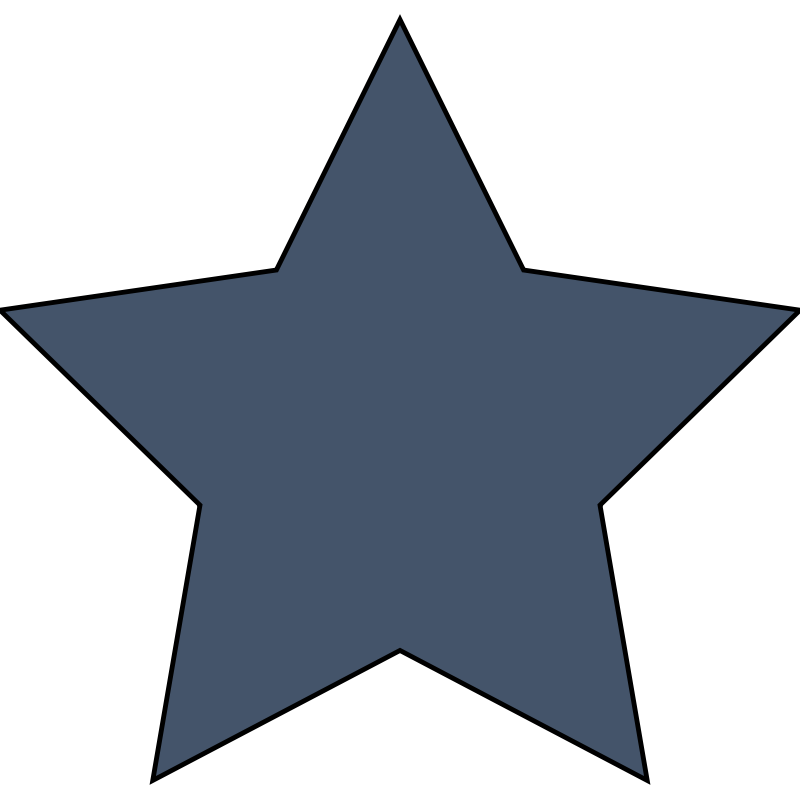 | 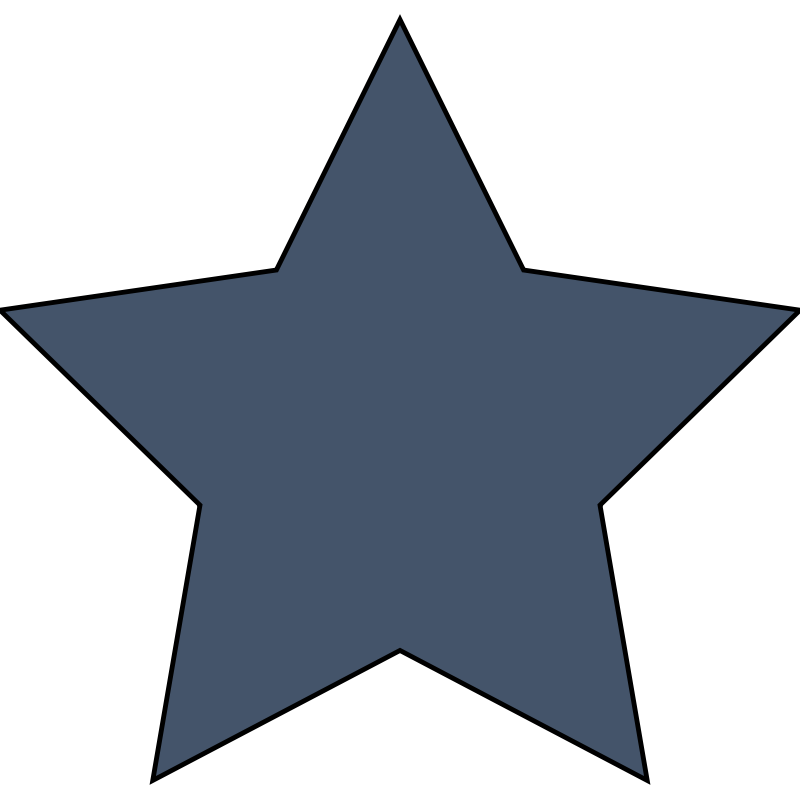 | 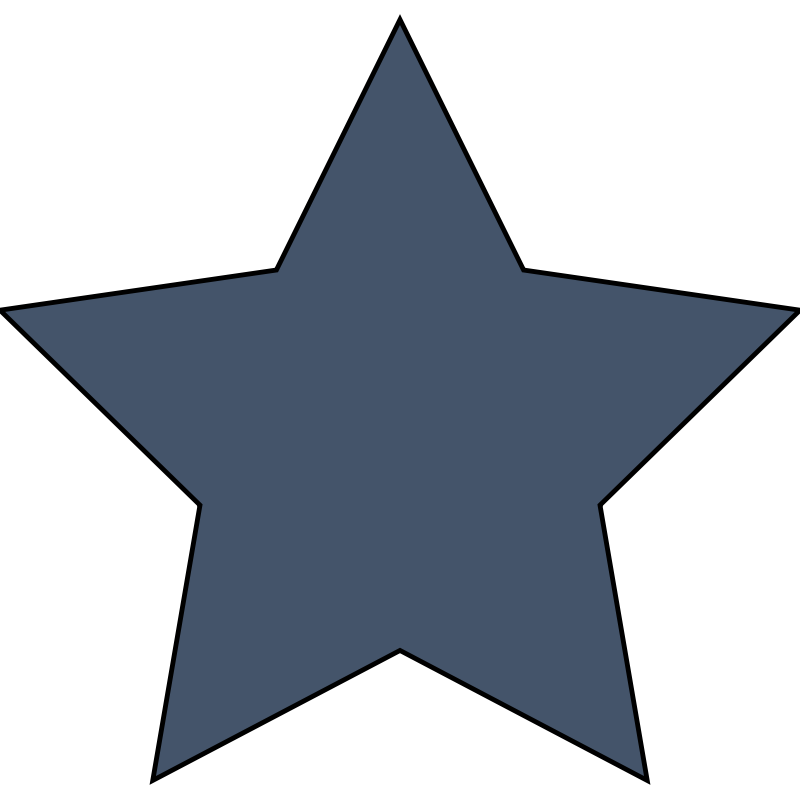 |
| Nithya Sukumar, 2021 | 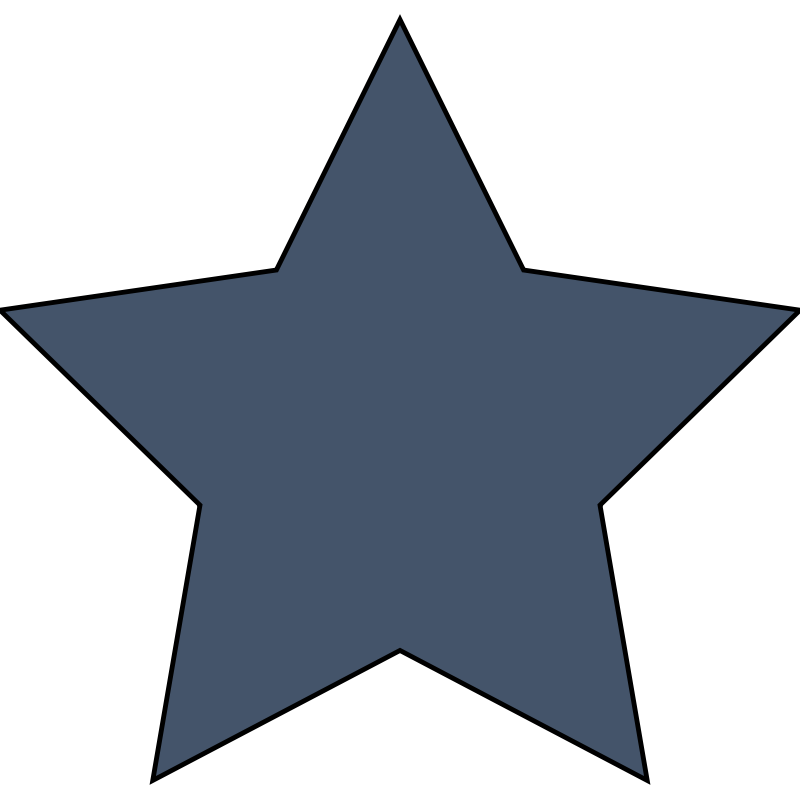 | 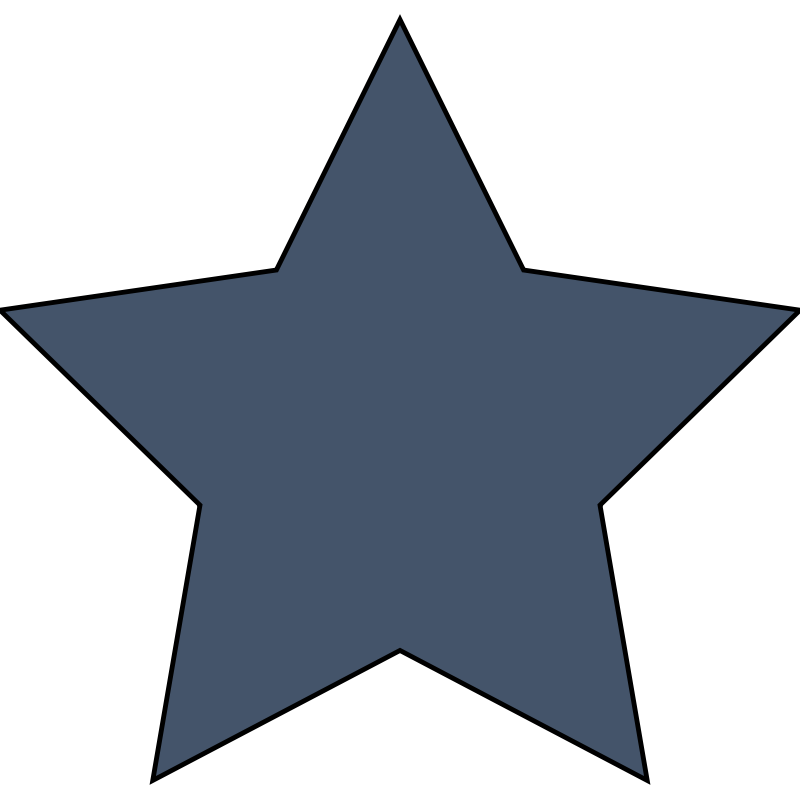 | 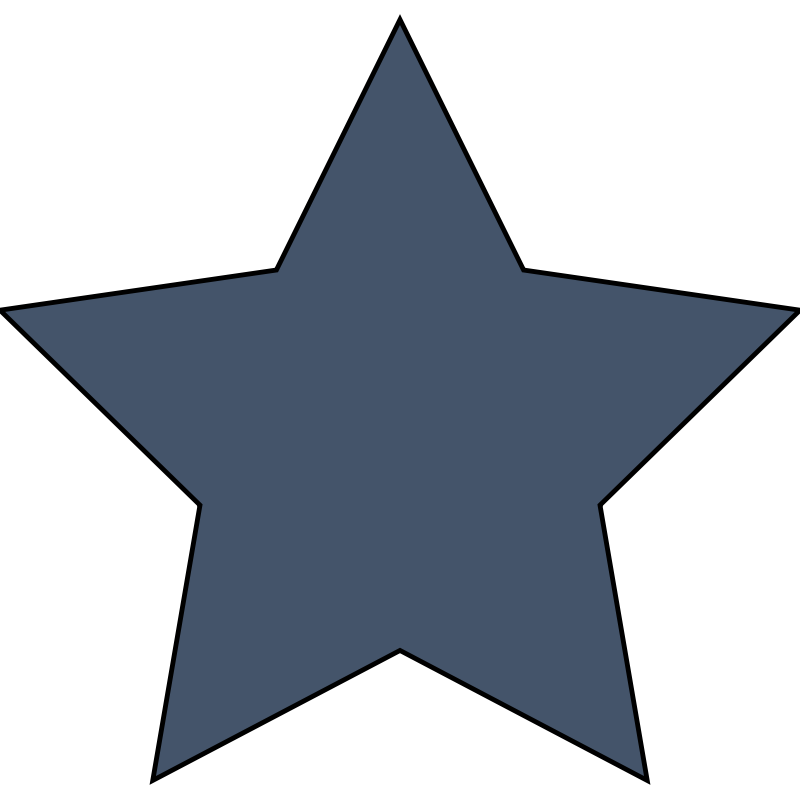 |  | 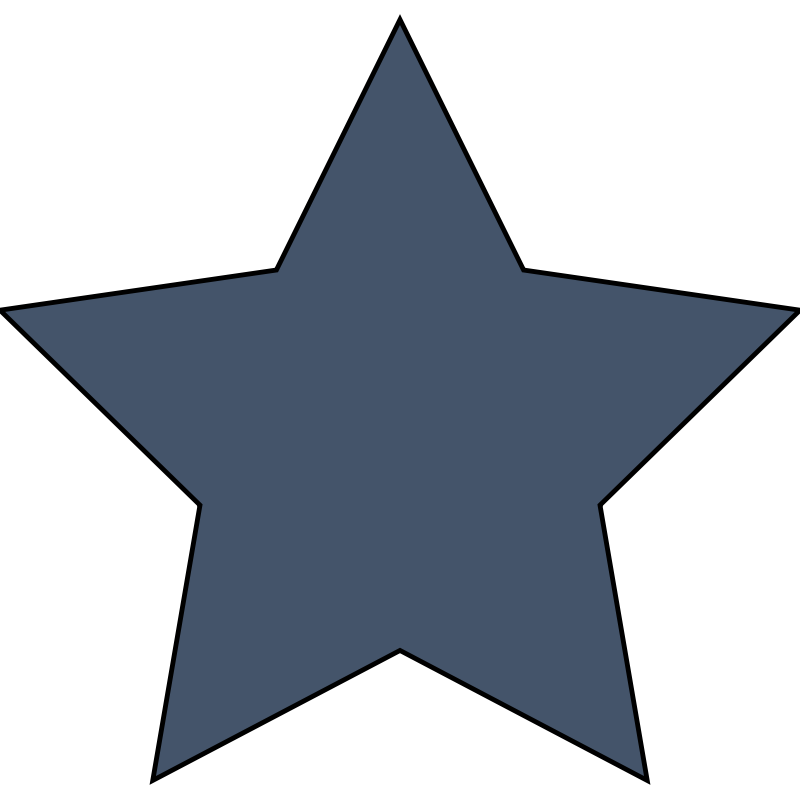 | 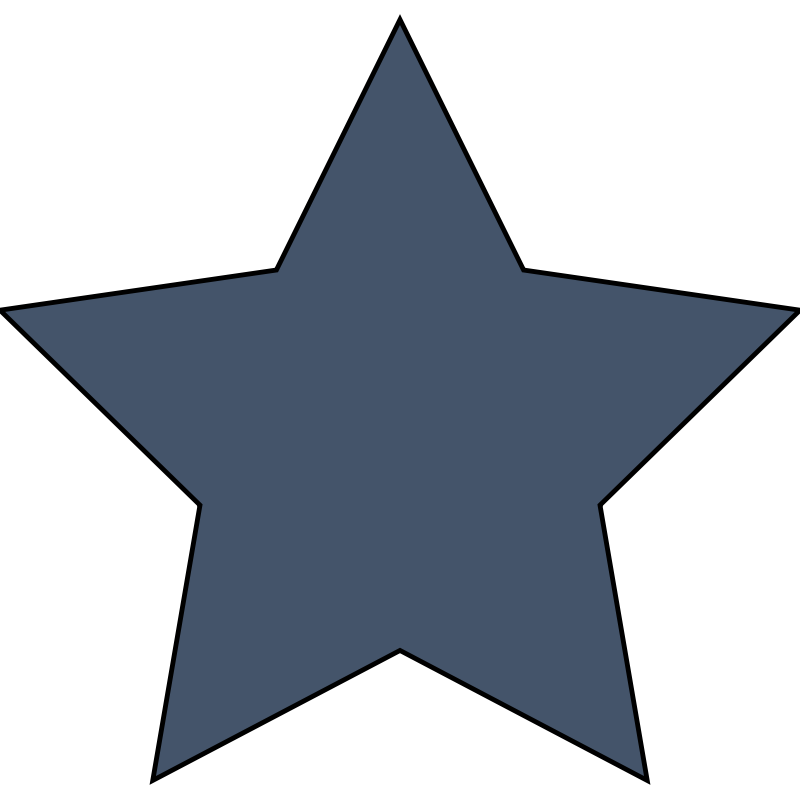 | 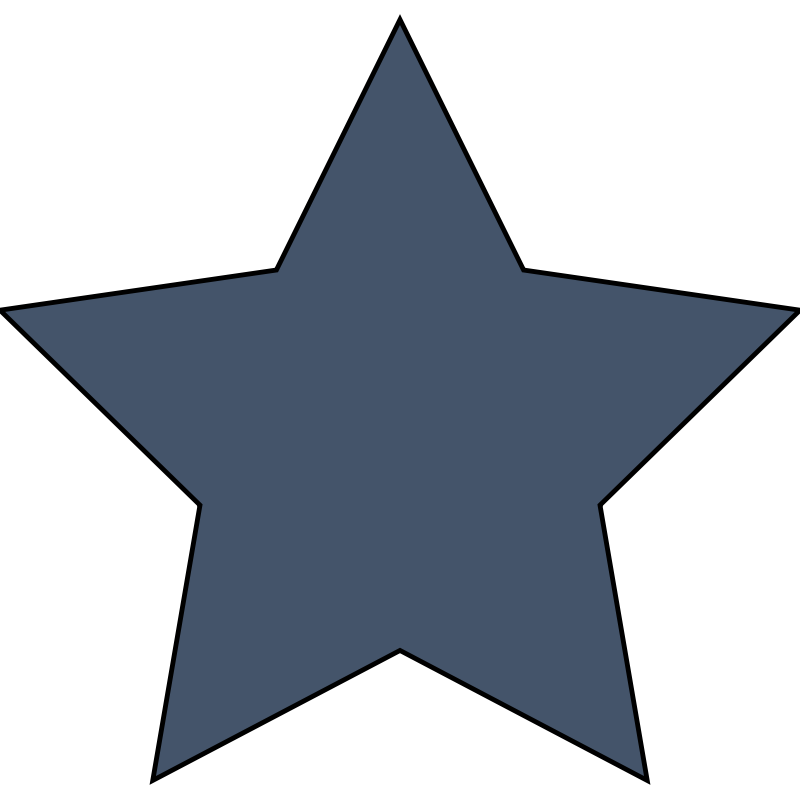 | 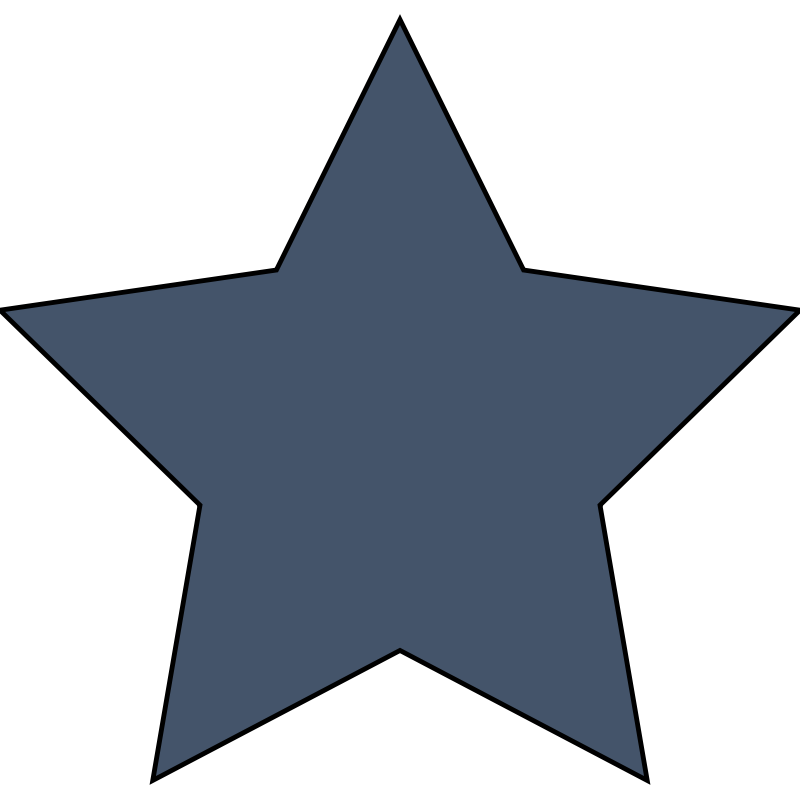 |

**Supplementary Table. 1-2 Newcastle–Ottawa Scale of cohort studies**

| Author | Case definition adequate | Representativeness of the cases | Selection of controls | Definition of control | Comparability of cases and controls | Ascertainment  of exposure | Same method  of cases and  controls | Non-response rate |
| --- | --- | --- | --- | --- | --- | --- | --- | --- |
| Nithya Sukumar, 2016 | 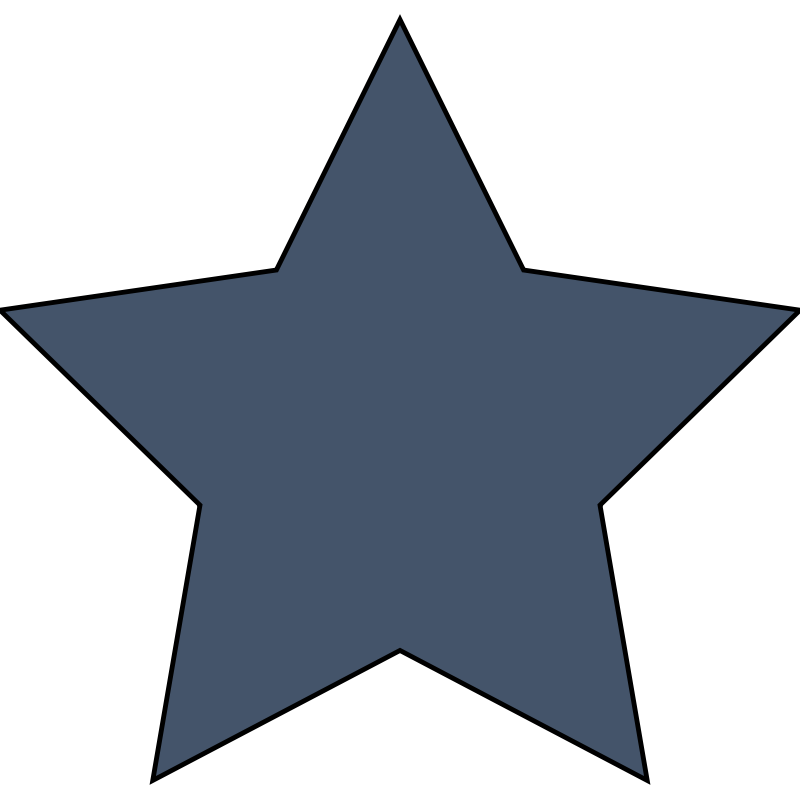 | 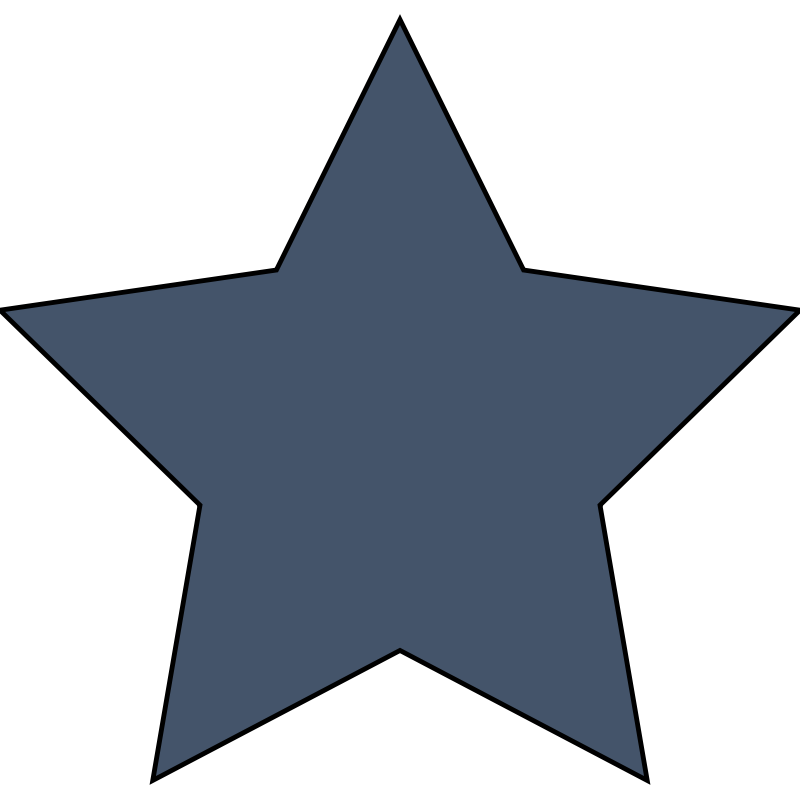 | 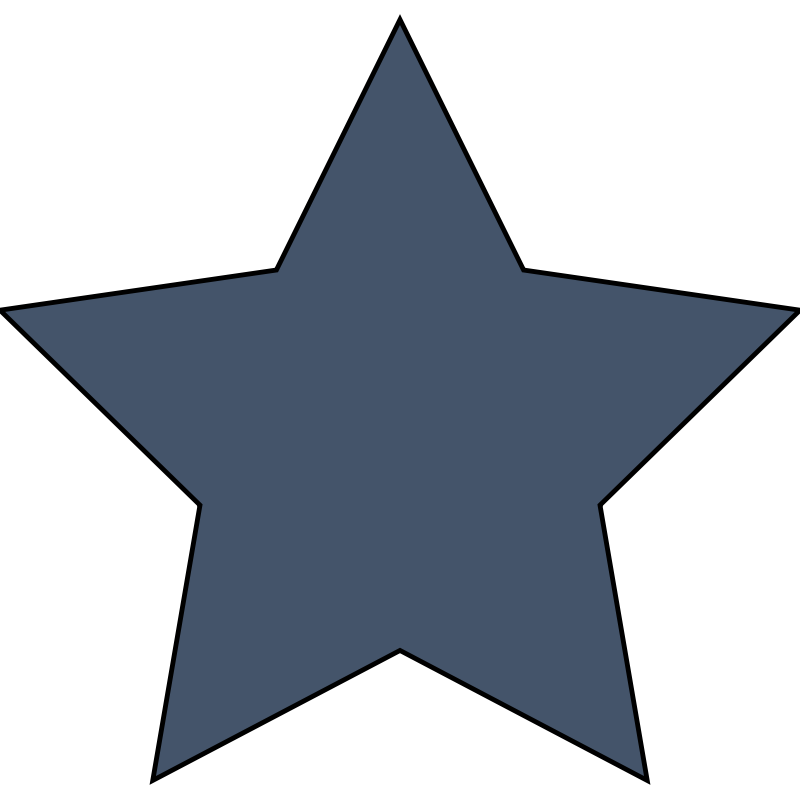 | 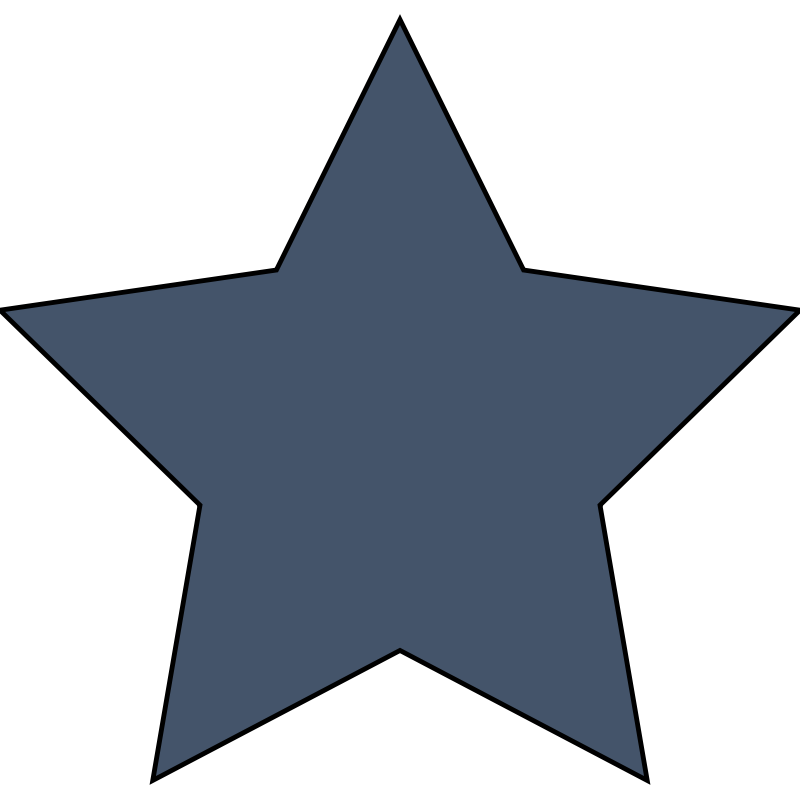 | 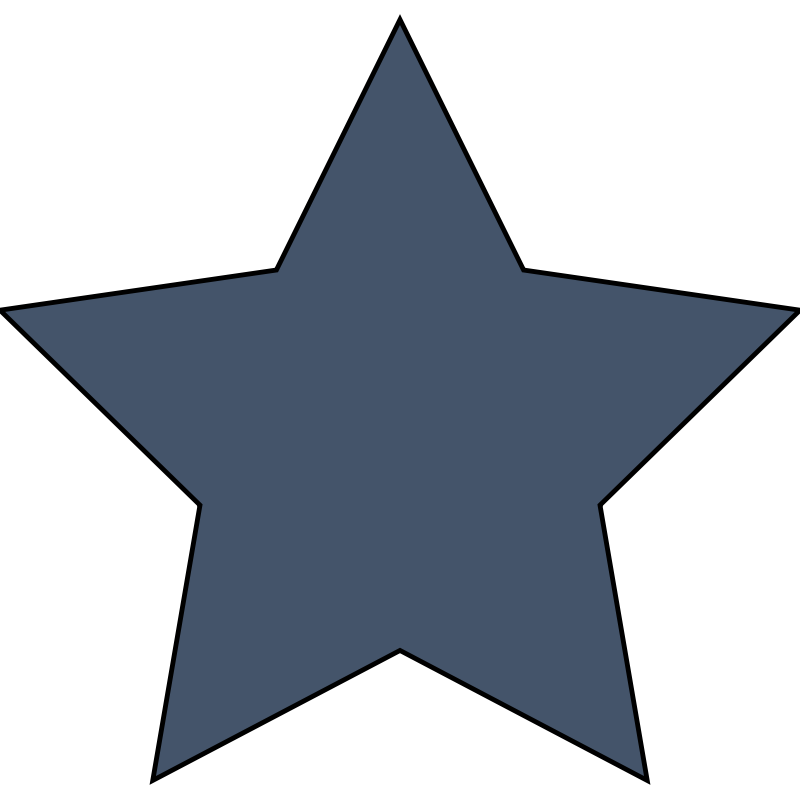 | 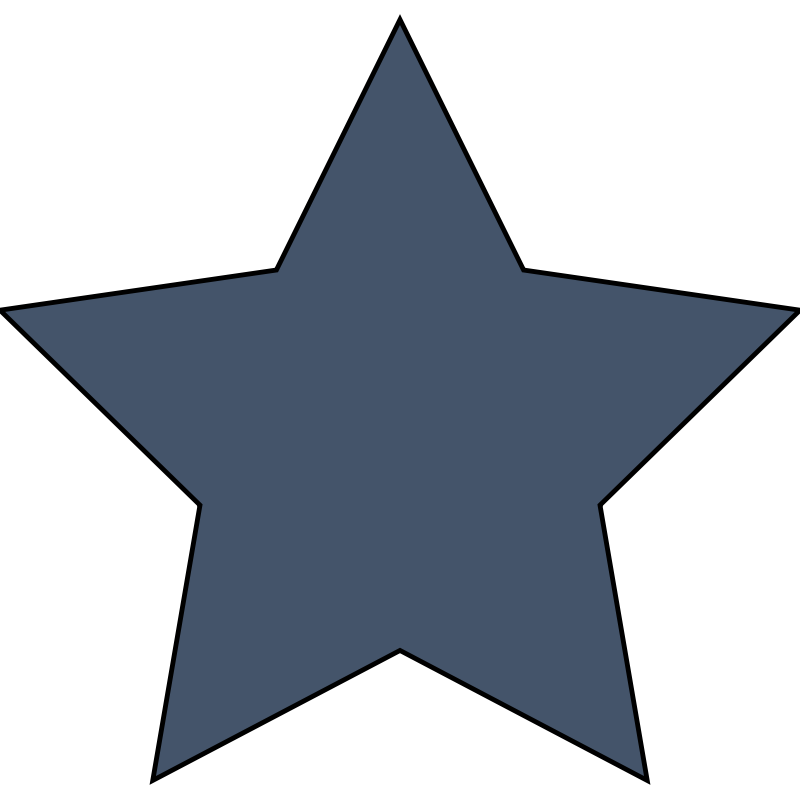 | 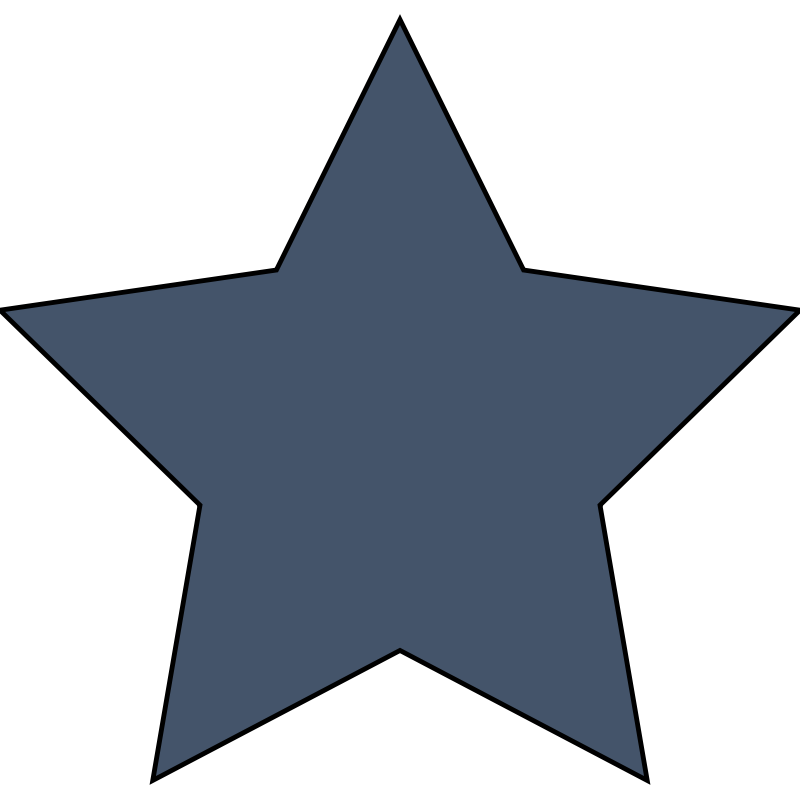 |  |
| Jun S Lai, 2018 | 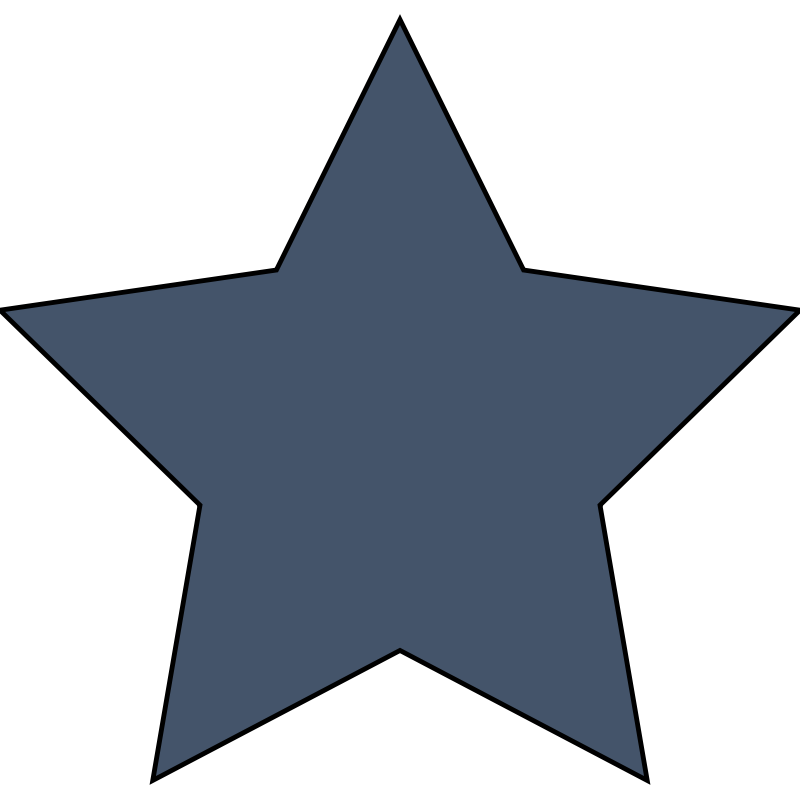 | 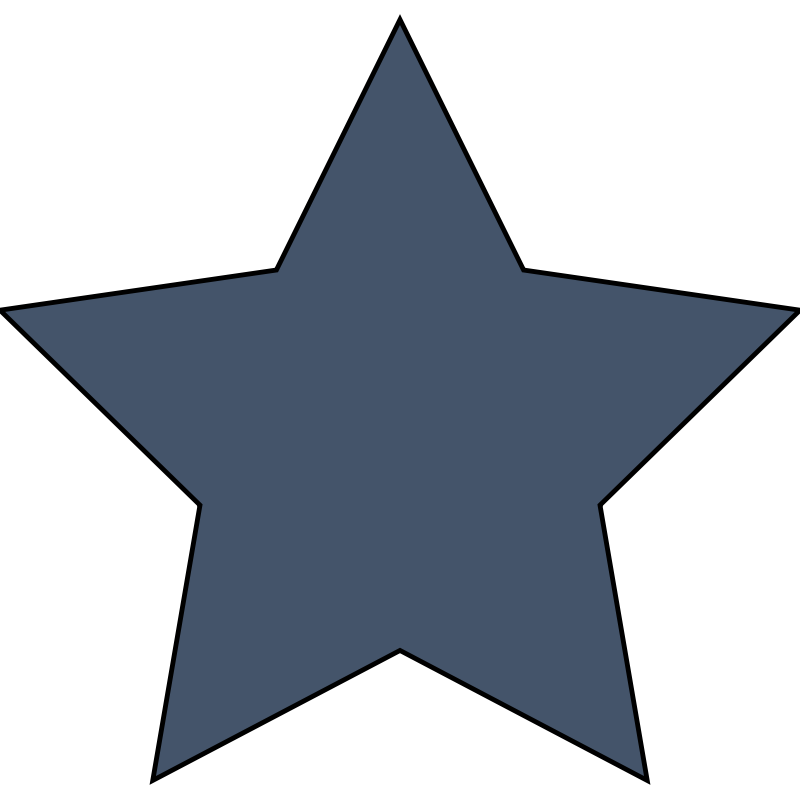 | 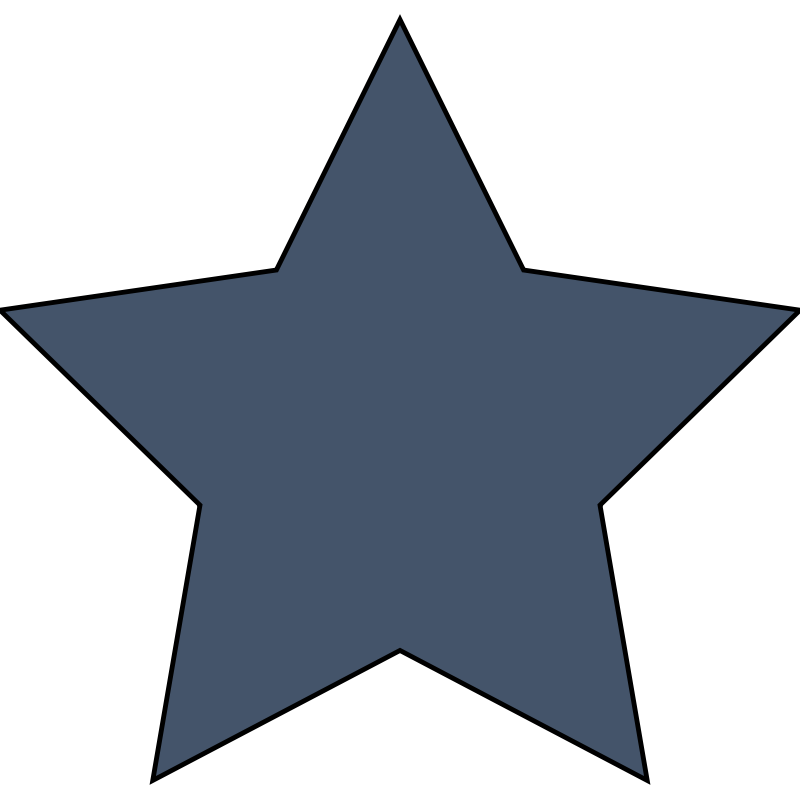 | 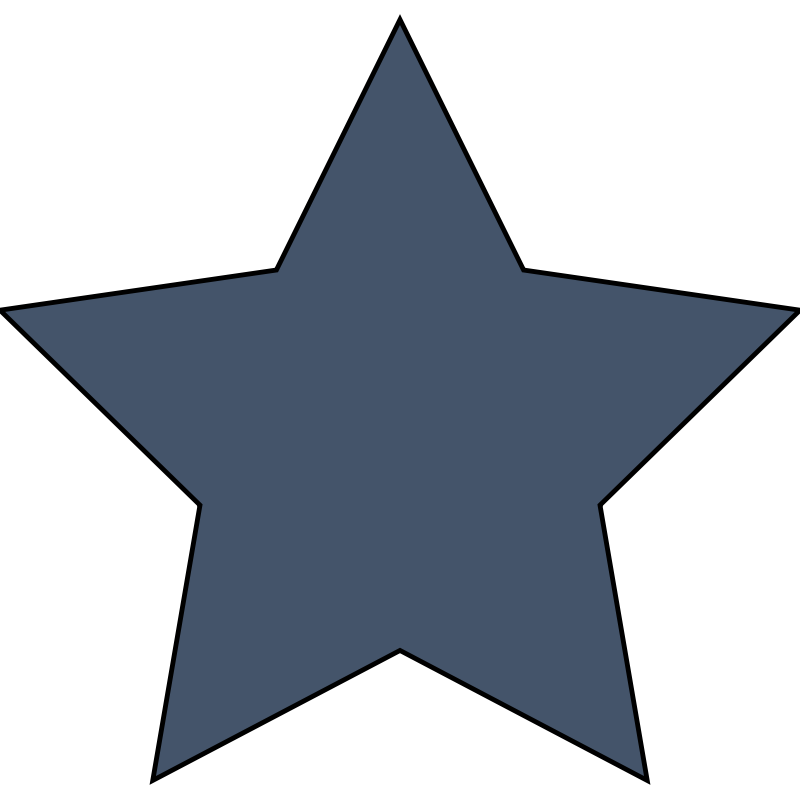 | 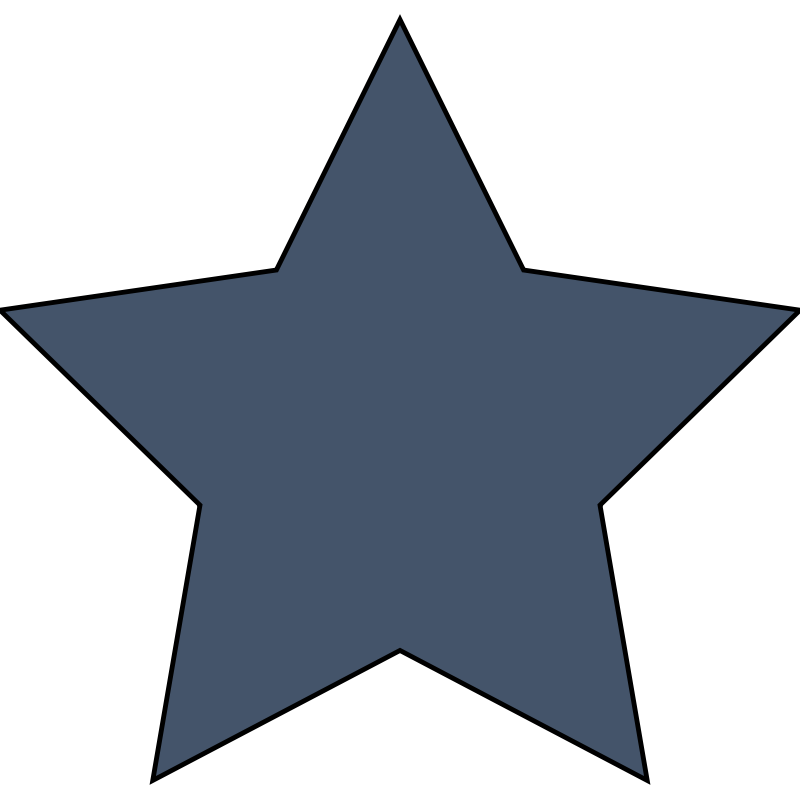 | 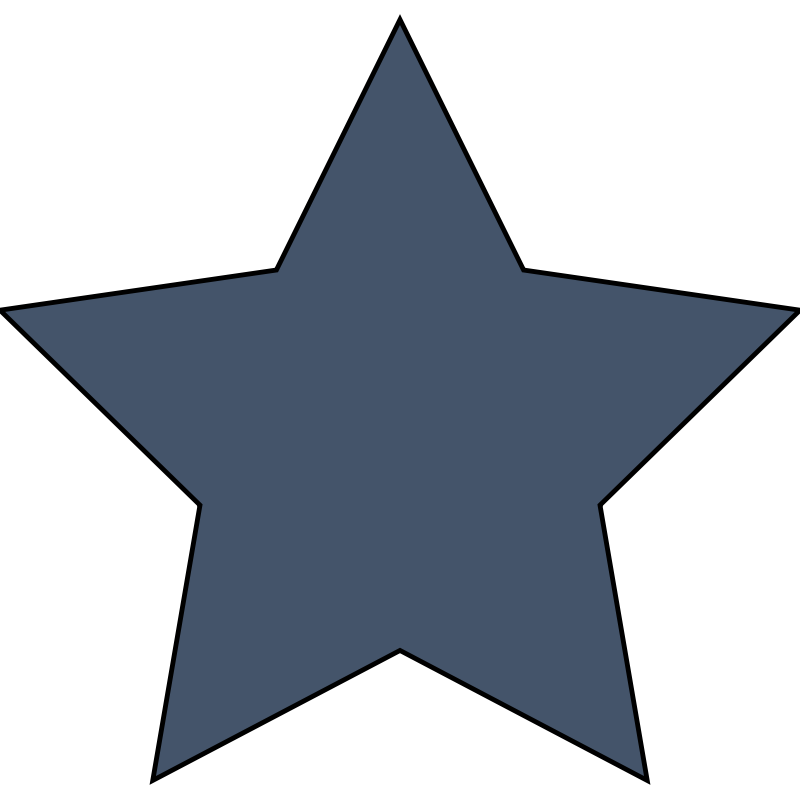 | 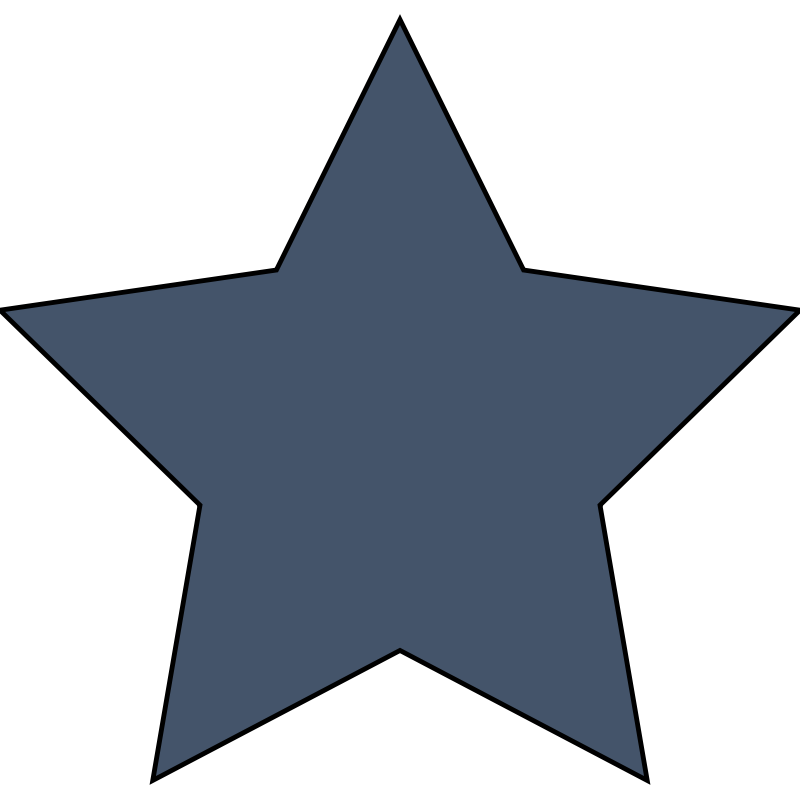 |  |
| Shuying Li, 2019 | 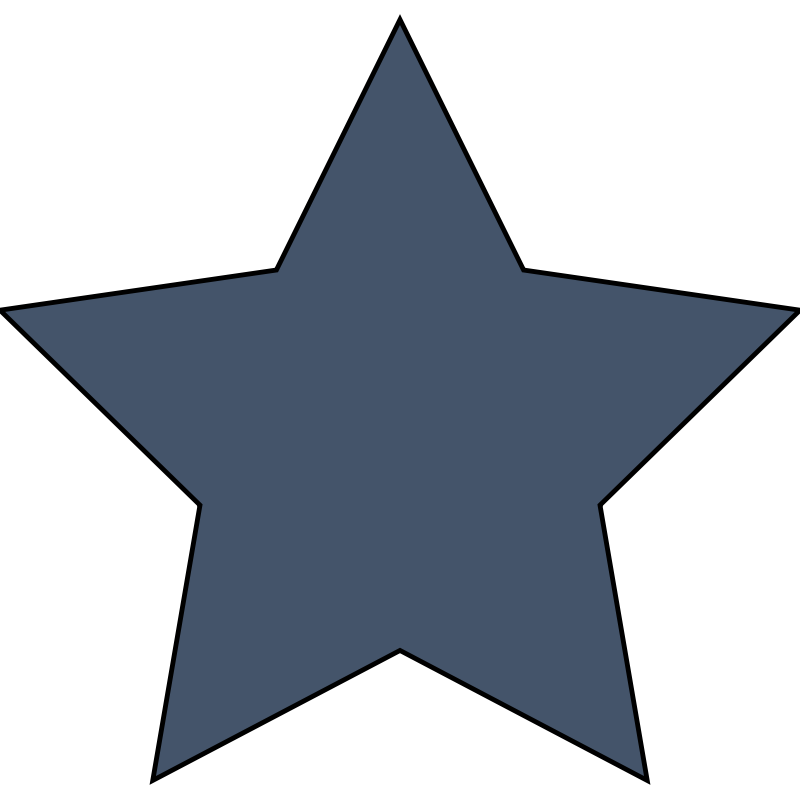 | 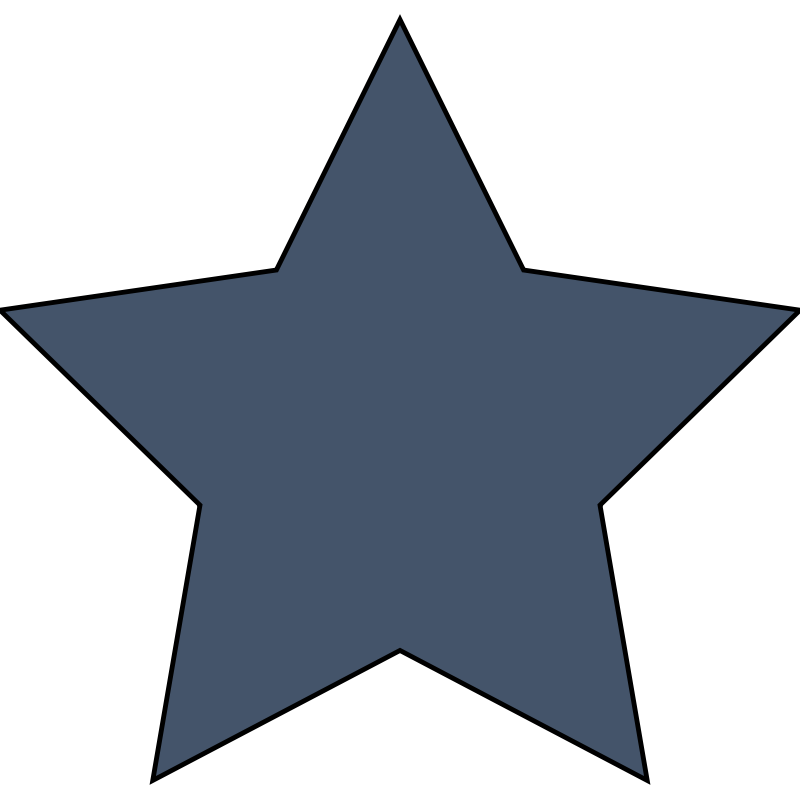 | 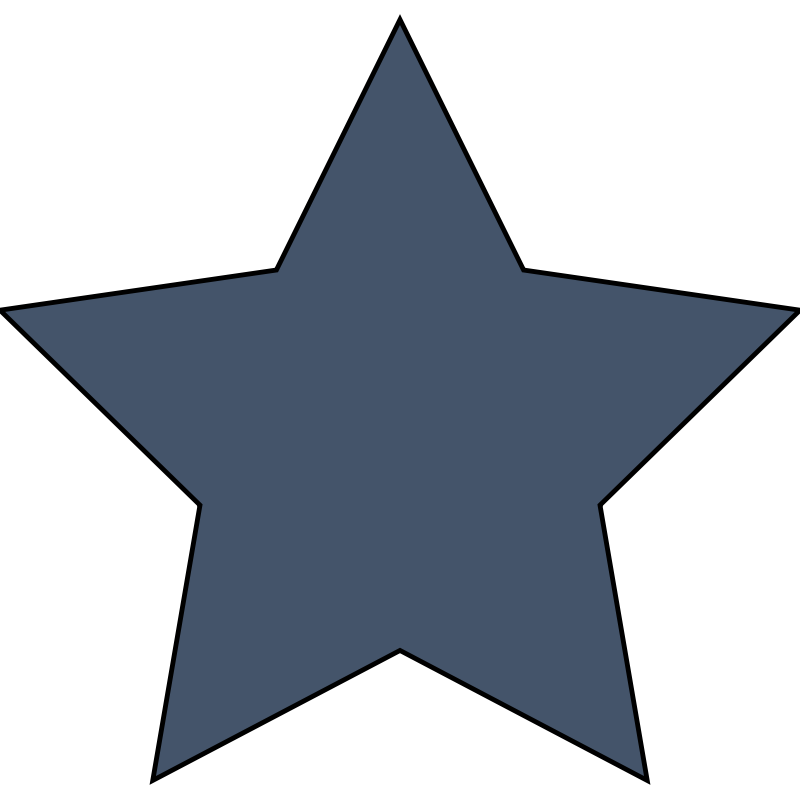 | 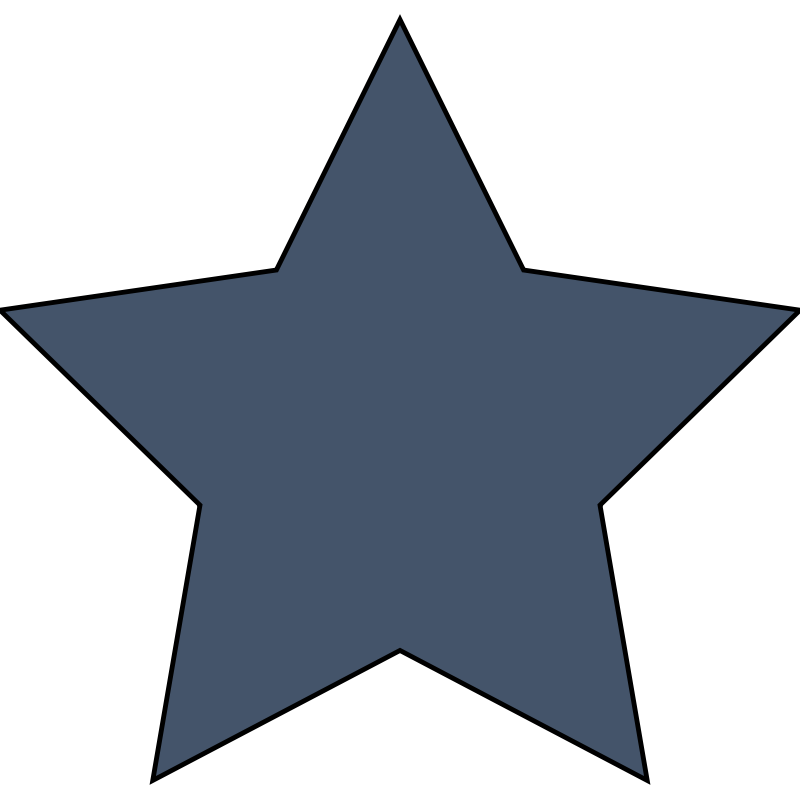 | 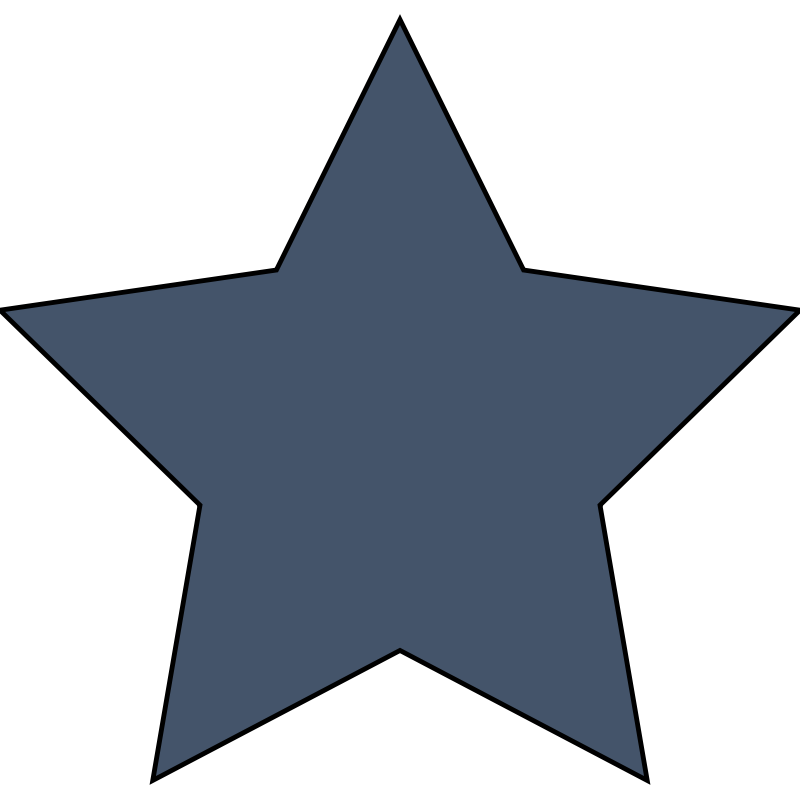 | 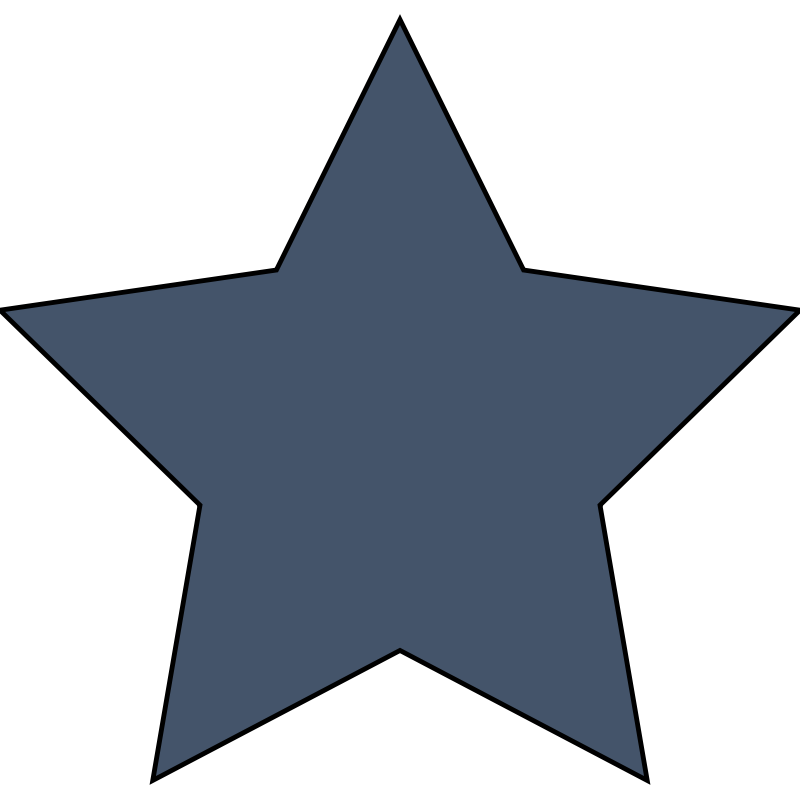 | 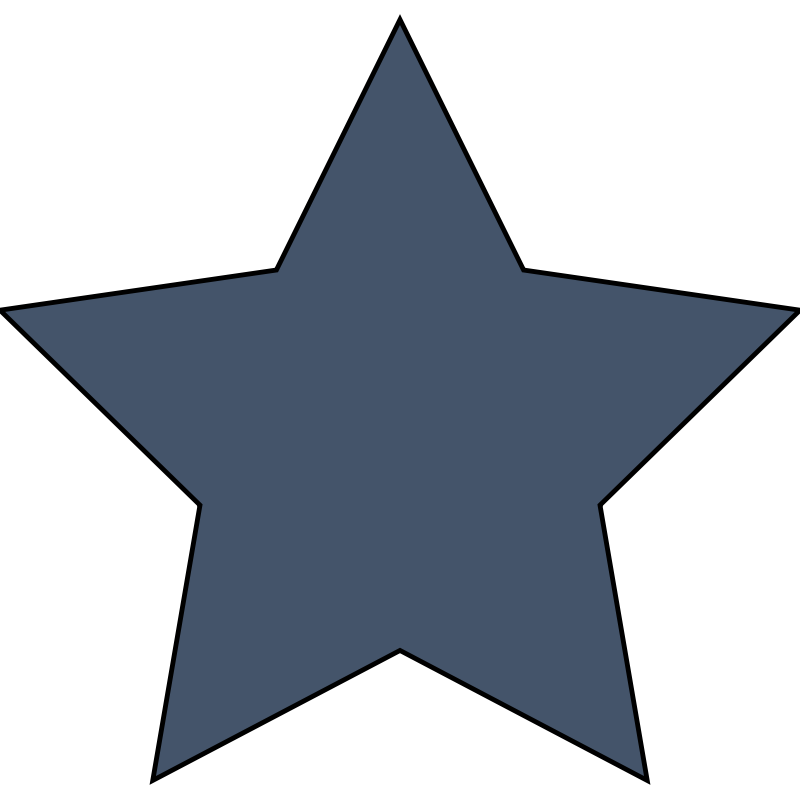 |  |
| Tanja, 2020 | 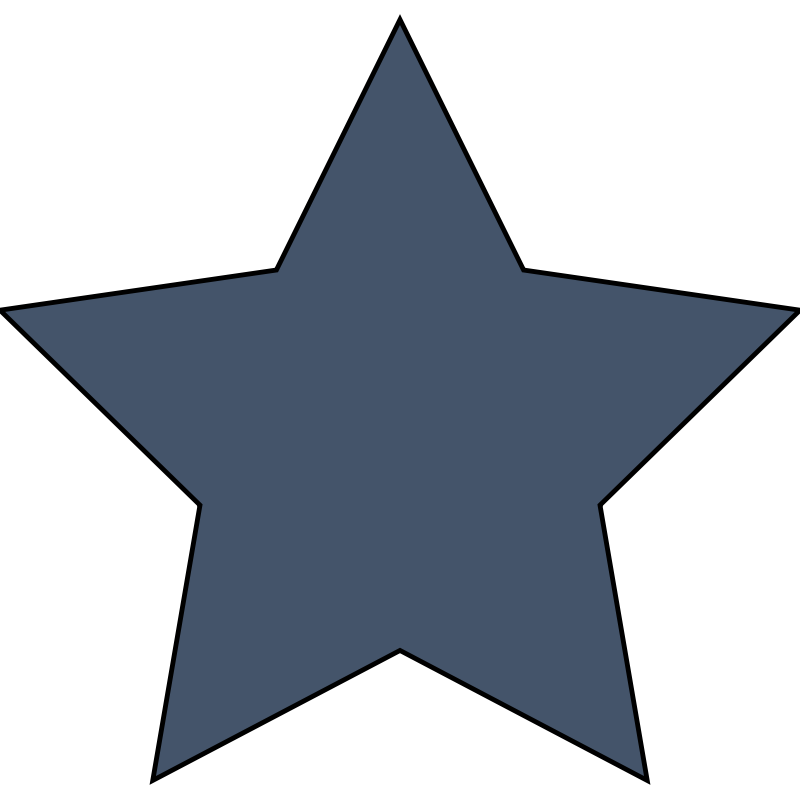 | 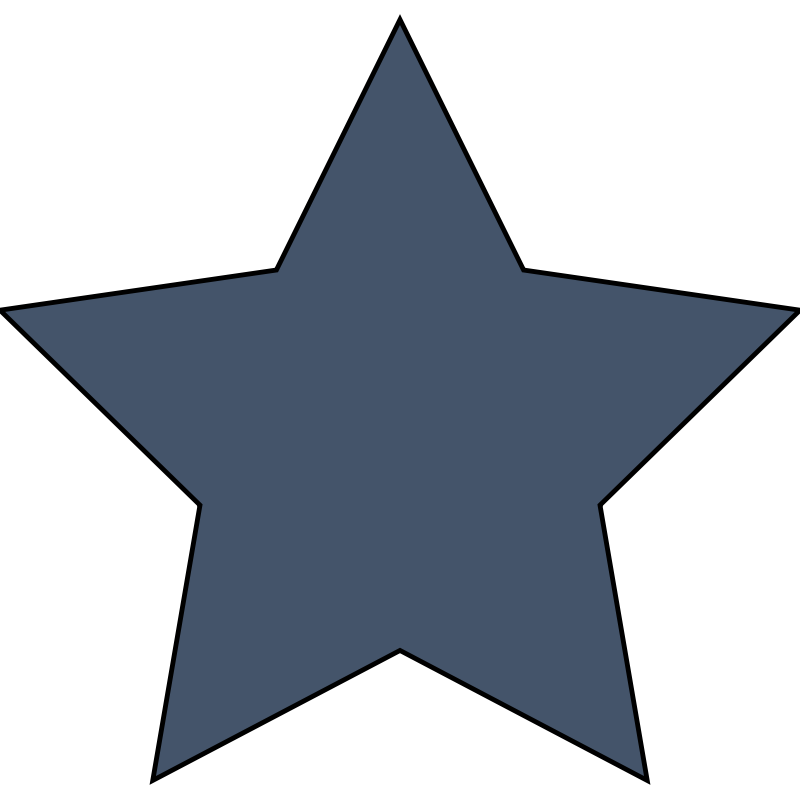 | 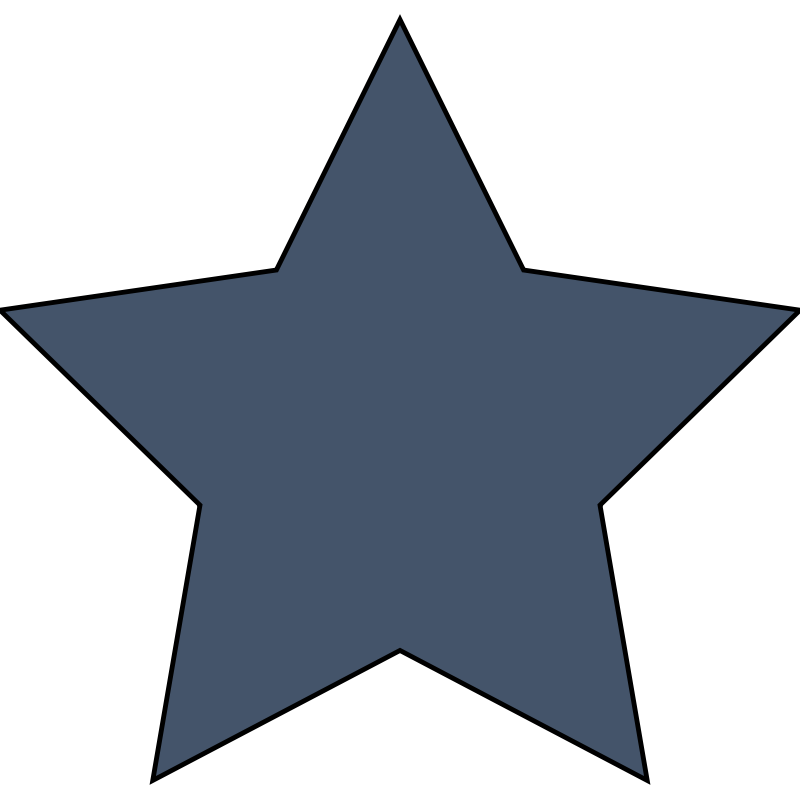 | 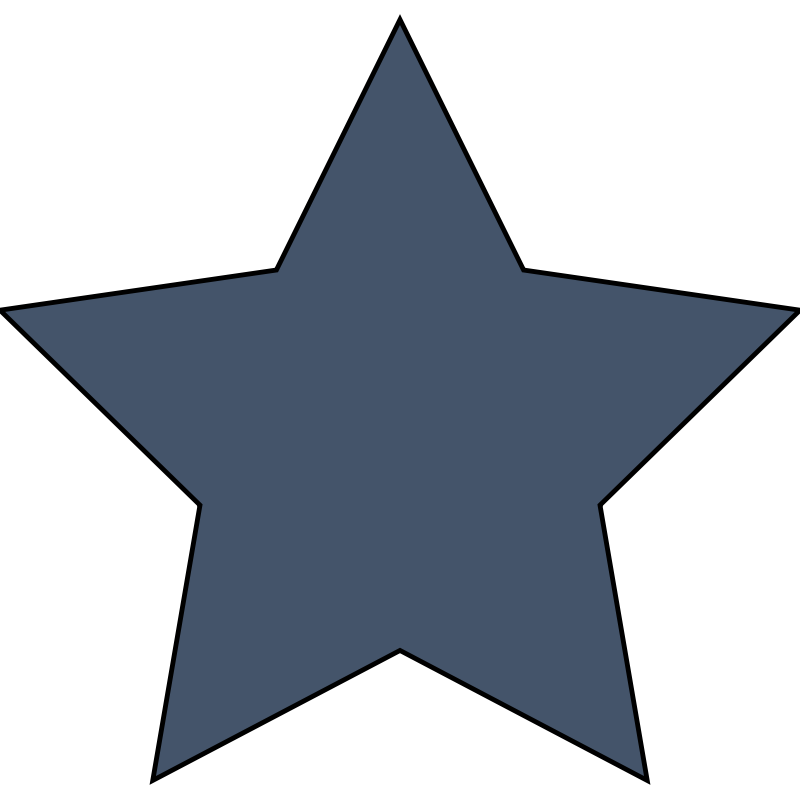 | 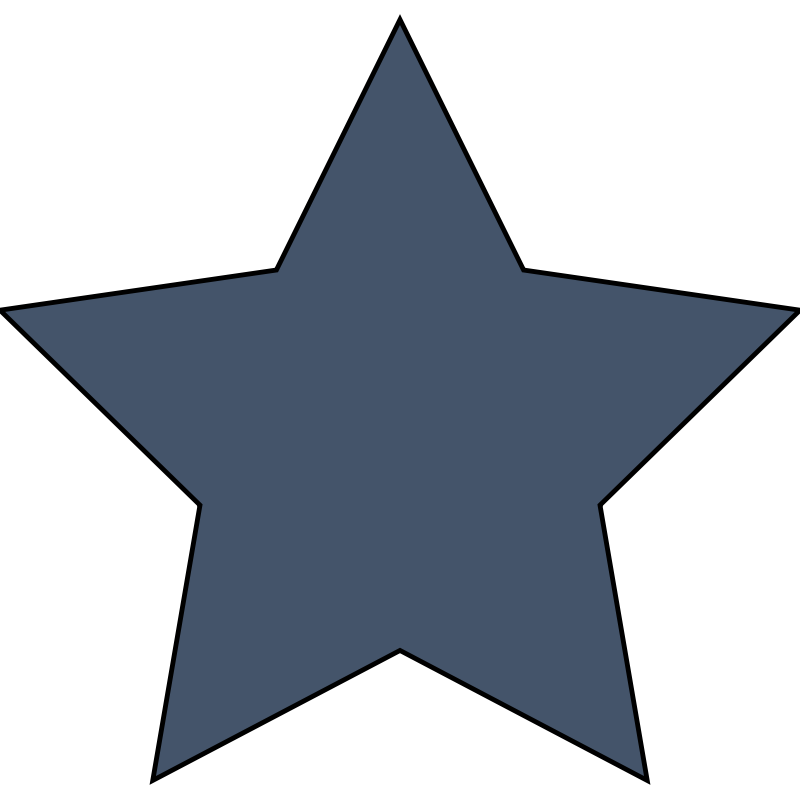 | 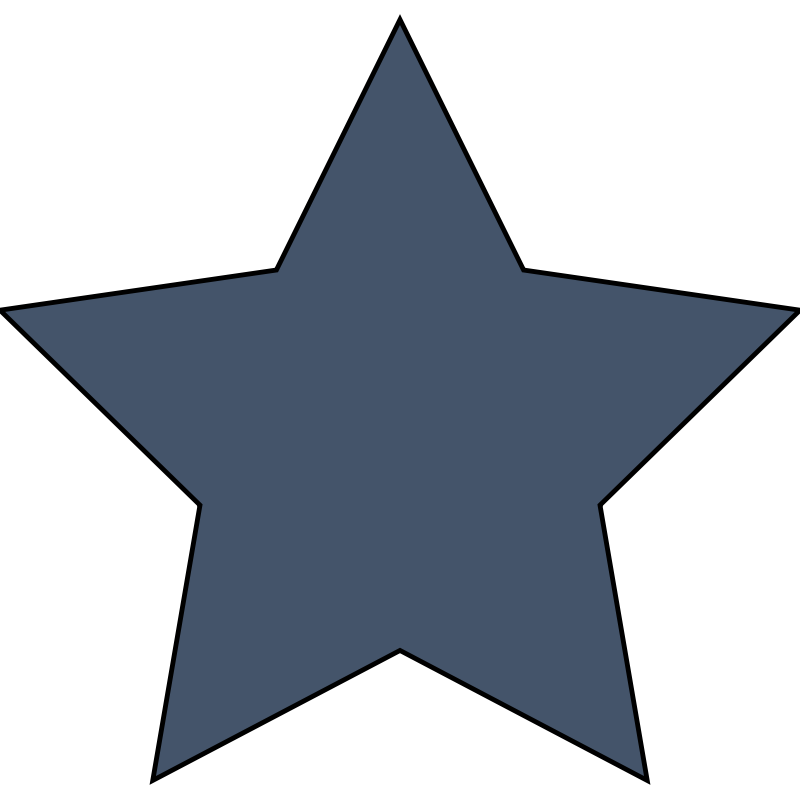 | 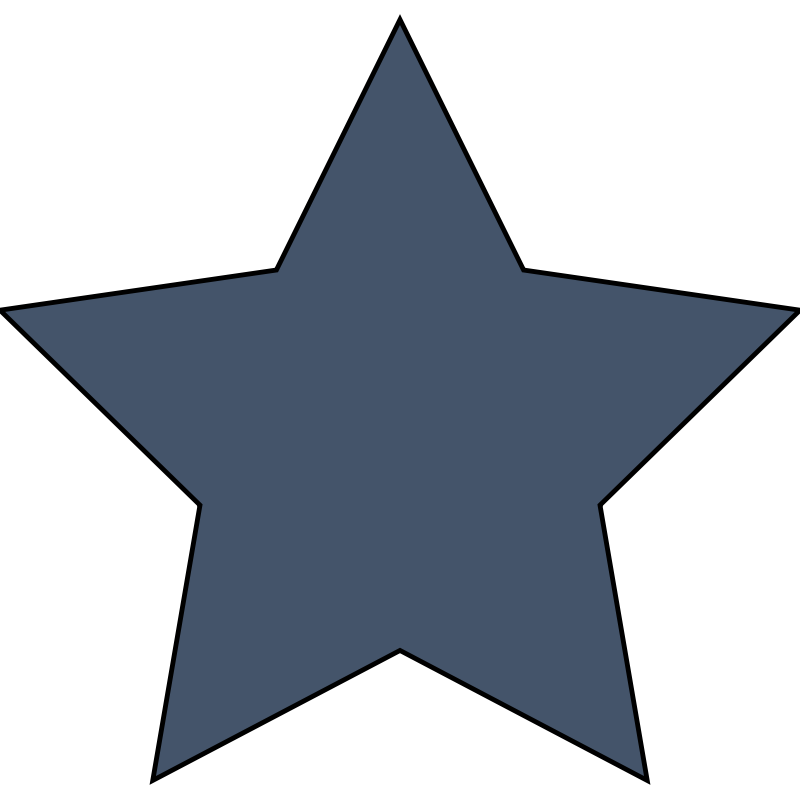 |  |
| Wang Shiqin, 2020 | 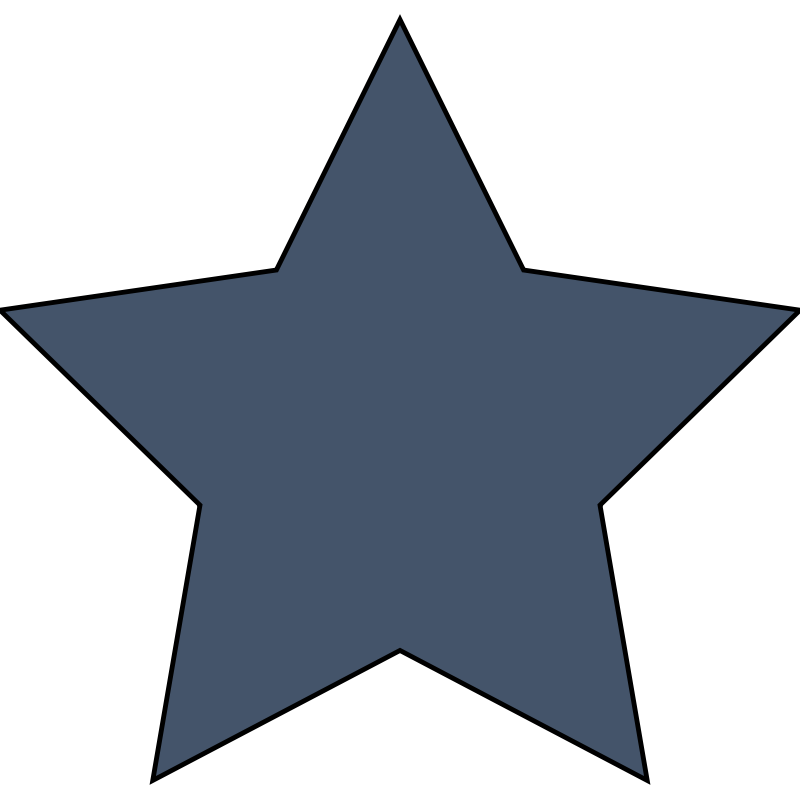 |  | 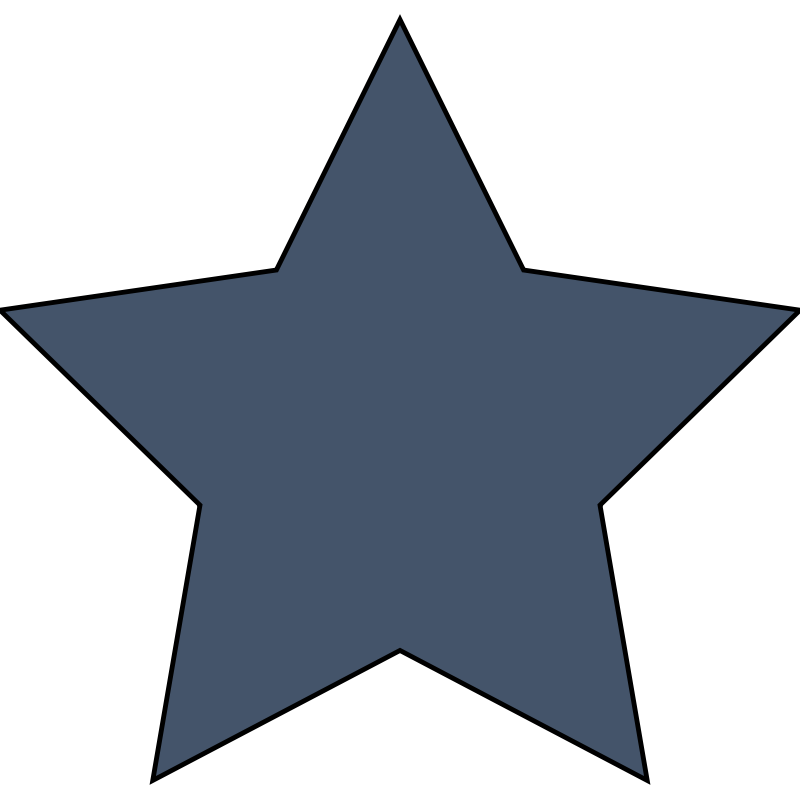 | 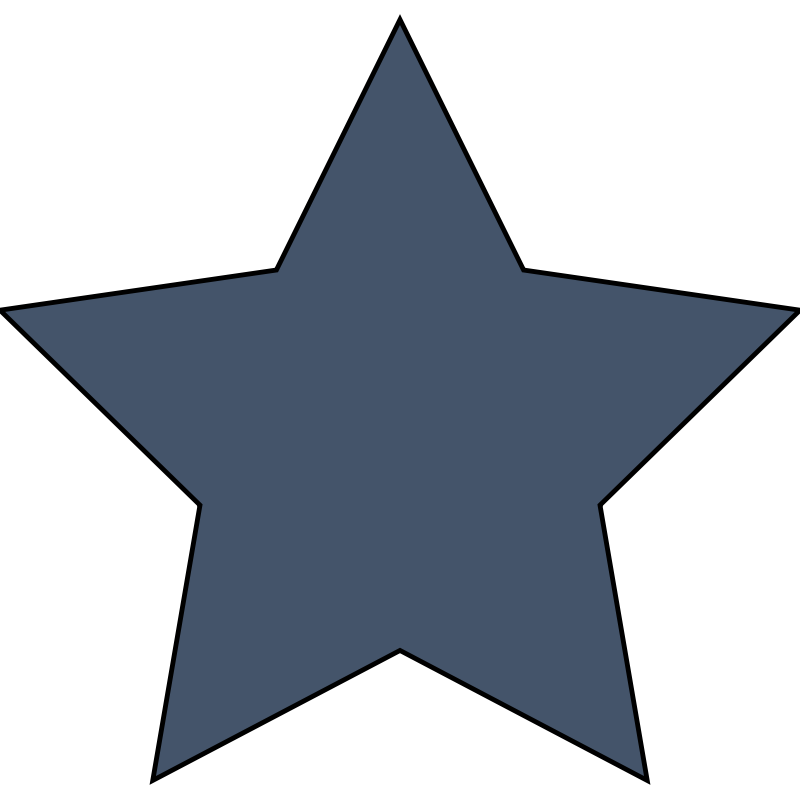 |  | 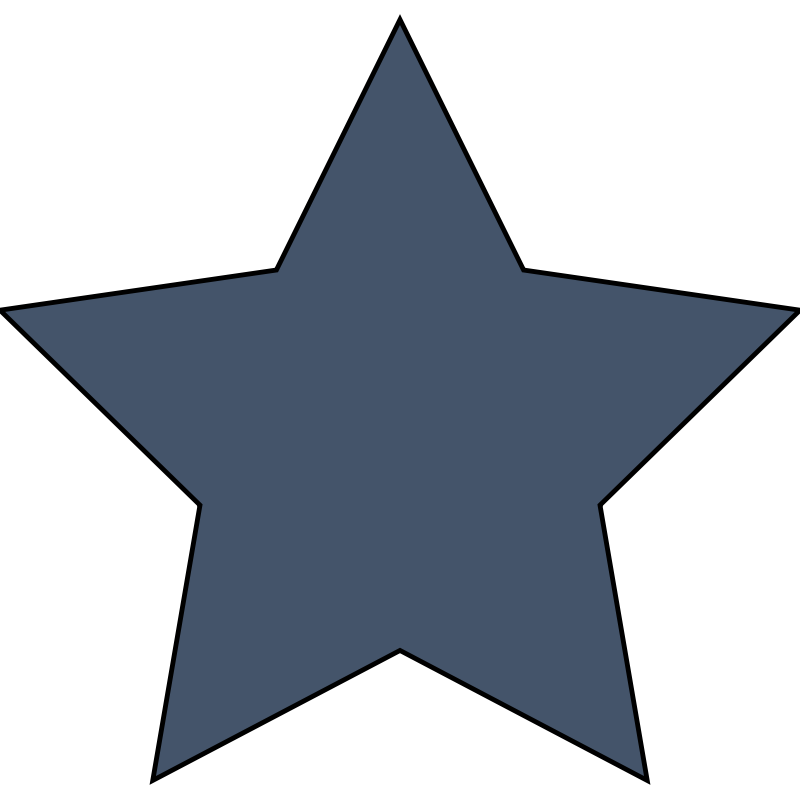 |  |  |
| Shuying Li, 2022 | 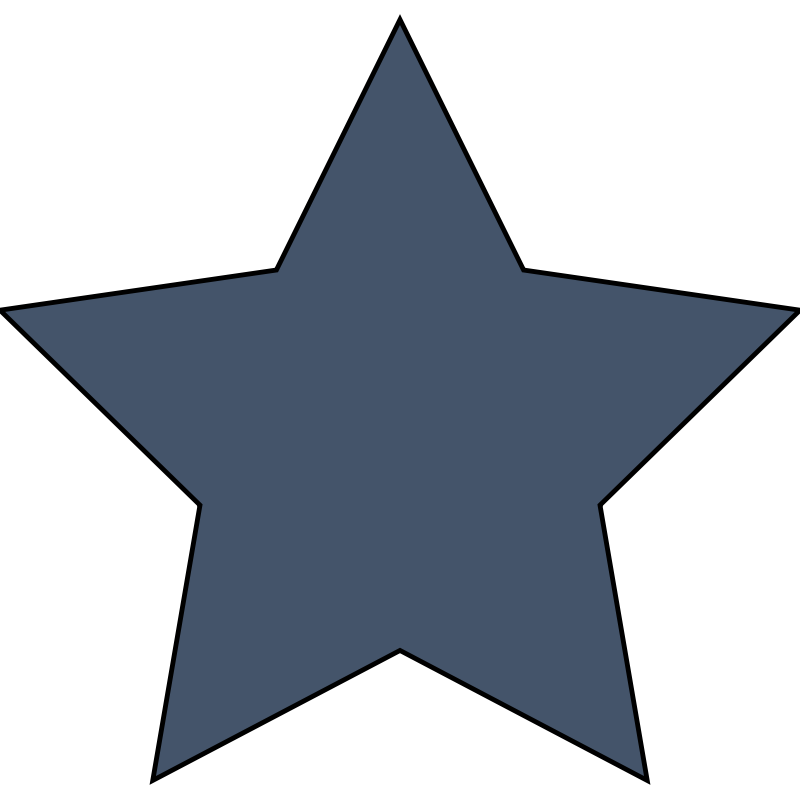 | 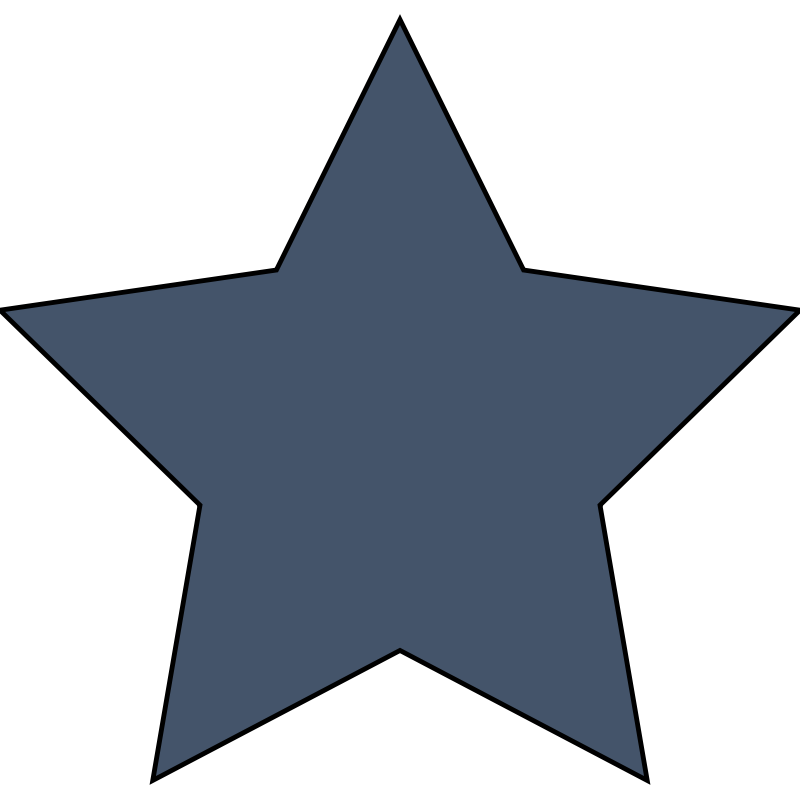 | 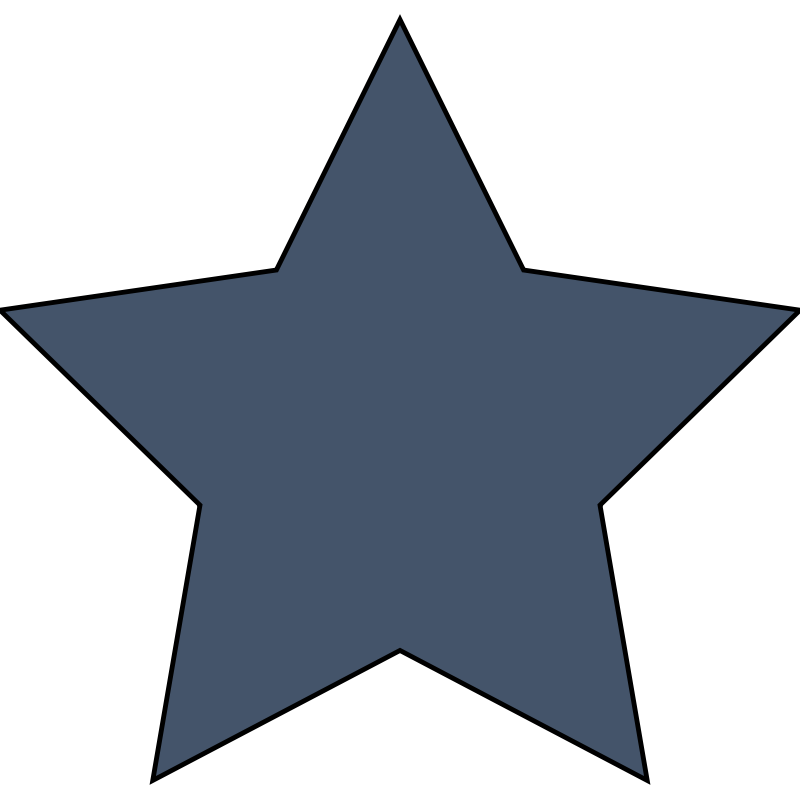 | 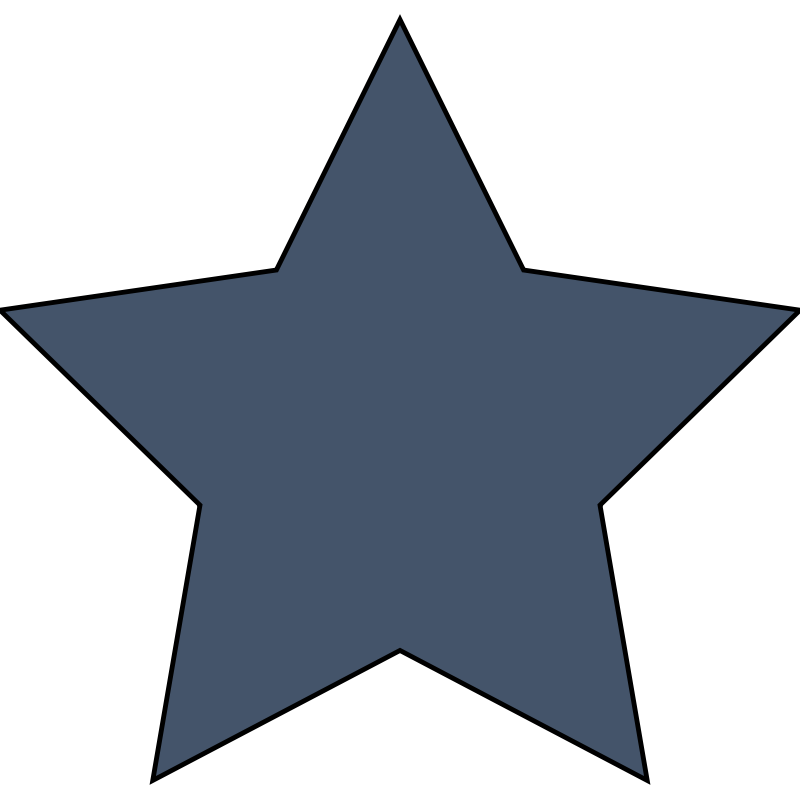 | 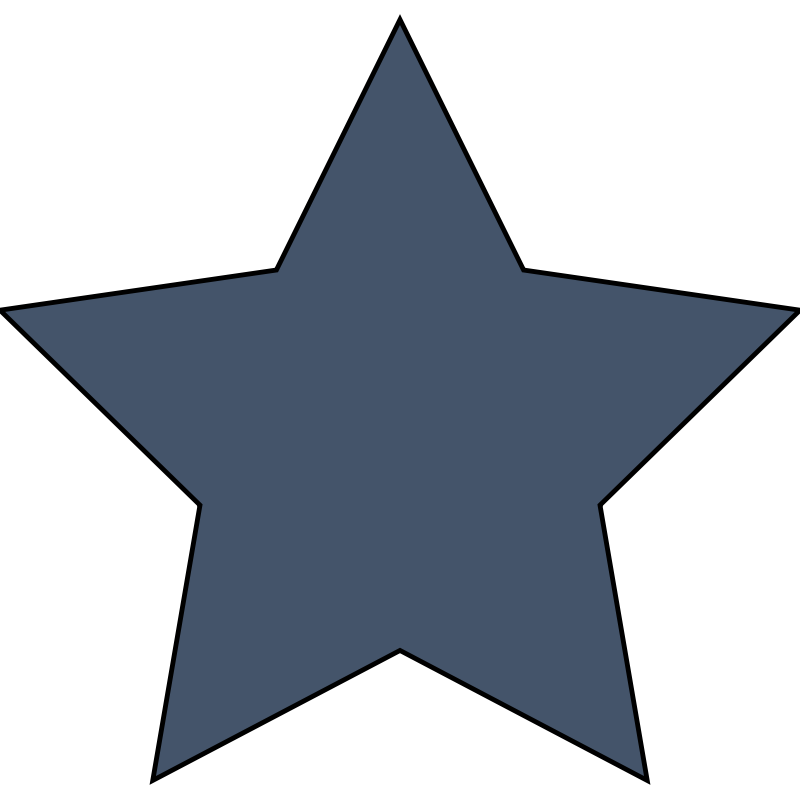 | 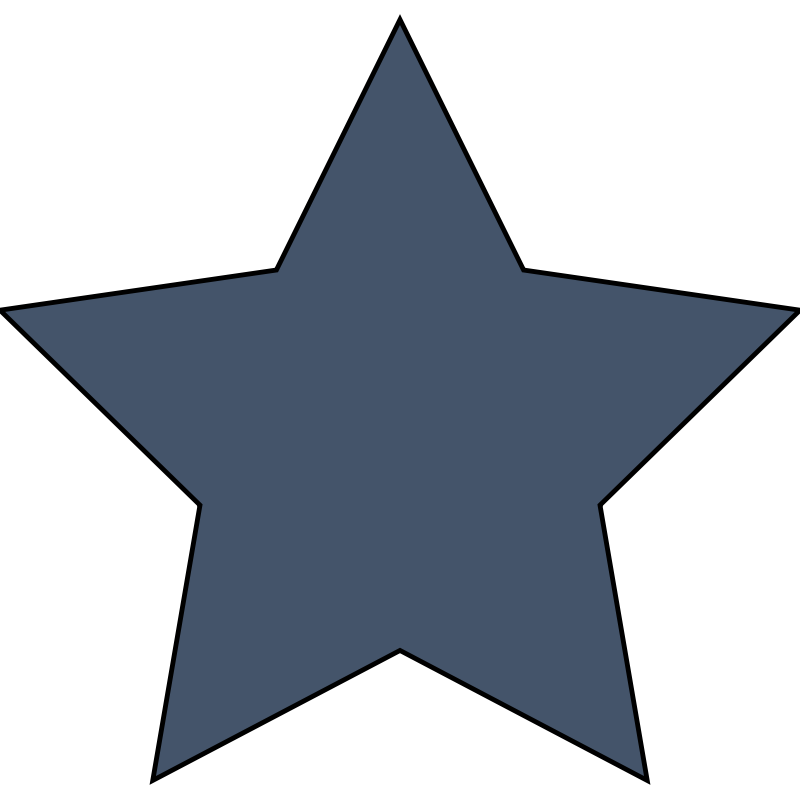 | 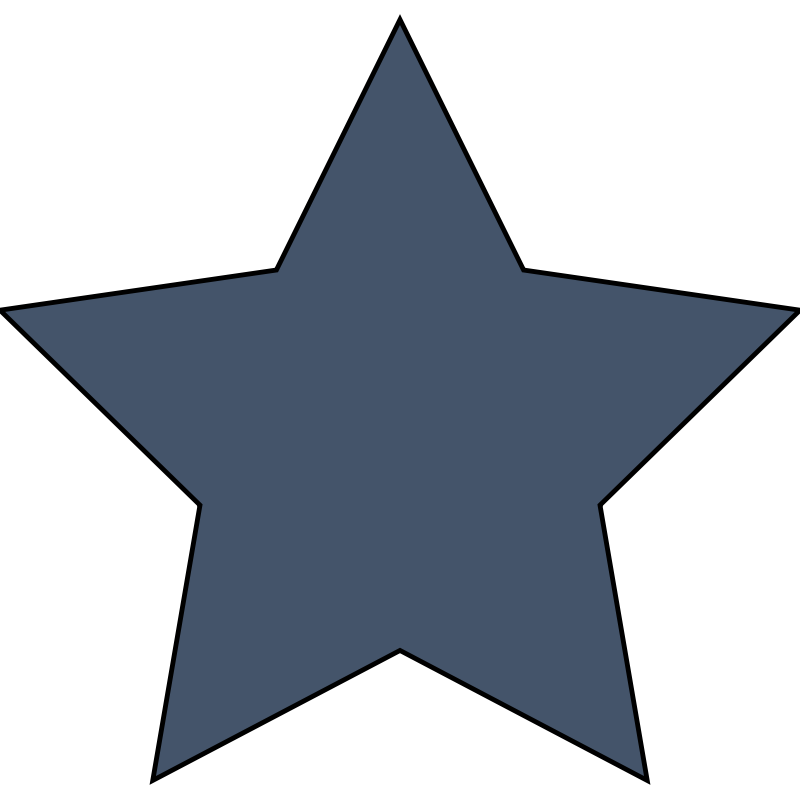 |  |
