# Supplementary material for: Vitamin B12 status and folic acid/vitamin B12 related to the risk of gestational diabetes mellitus in pregnancy: a systematic review and meta-analysis of observational studies
Source: BMC Pregnancy Childbirth. 2022 Jul 23;22:587. doi: 10.1186/s12884-022-04911-9 (PMC9308279; doi:10.1186/s12884-022-04911-9)
Supplement: Supplementary file 2 — Additional file 2: Supplementary Table. 2. Assessment of the Meta-Evidence of Cohort Studies for NutriGrade. [file 12884_2022_4911_MOESM2_ESM.docx]

**Supplementary Table. 2 Assessment of the Meta-Evidence of Cohort Studies for NutriGrade**

| **Item** | **Scoring Criteria** | **Score** |
| --- | --- | --- |
| Risk of bias/ study quality/ study limitations (2 P) | Newcastle–Ottawa Scale（mean）= 6.25 | 1 |
| Precision (1 P) | ≥500 events and the 95% CI excludes the null value | 1 |
| Heterogeneity (1 P) | Heterogeneity is observed，I2 ≥40% | 0.3 |
| Directness (1 P） | No important differences in the population or intervention | 1 |
| Publication bias (1 P) | ＜5 studies | 0 |
| Funding bias (1 P) | Academic institutions， research institutions | 1 |
| Effect size (2 P) | OR，＞1.20–2 | 1 |
| Dose-response (1 P) | No dose-response analysis | 0 |
| Total |  | 5.3 |

4 categories to judge the meta-evidence: high, moderate, low, and very low. ≥8 points (high meta-evidence); 6–7.99 points (moderate meta-evidence); 4–5.99 points (low meta evidence); and 0–3.99 points (very low meta-evidence).
