# Supplementary material for: Vitamin B12 status and folic acid/vitamin B12 related to the risk of gestational diabetes mellitus in pregnancy: a systematic review and meta-analysis of observational studies
Source: BMC Pregnancy Childbirth. 2022 Jul 23;22:587. doi: 10.1186/s12884-022-04911-9 (PMC9308279; doi:10.1186/s12884-022-04911-9)
Supplement: Supplementary file 3 — Additional file 3: Supplementary Table. 3 PICOS framework. [file 12884_2022_4911_MOESM3_ESM.docx]

**Supplementary Table. 3 PICOS framework**

|  | **Inclusion** | **Exclusion** |
| --- | --- | --- |
| Patient | Pregnant women | Pre-existing diabetes  Supplement of vitamin B12 |
| Exposure | Vitamin B12 deficiency |  |
| Control | Vitamin B12 sufficiency |  |
| Outcome | Diagnosed with GDM |  |
| Study | Cohort, case-control, cross-section |  |
